# 1-Ethyl-3-methylimidazolium Acetate as a Reactive Solvent for Elemental Sulfur and Poly(sulfur nitride)

**DOI:** 10.1021/acs.jpcb.4c01536

**Published:** 2024-06-01

**Authors:** Julian Radicke, Karsten Busse, Vanessa Jerschabek, Haleh Hashemi Haeri, Muhammad Abu Bakar, Dariush Hinderberger, Jörg Kressler

**Affiliations:** Department of Chemistry, Martin Luther University Halle-Wittenberg, von-Danckelmann-Platz 4, D-06120 Halle (Saale), Germany

## Abstract

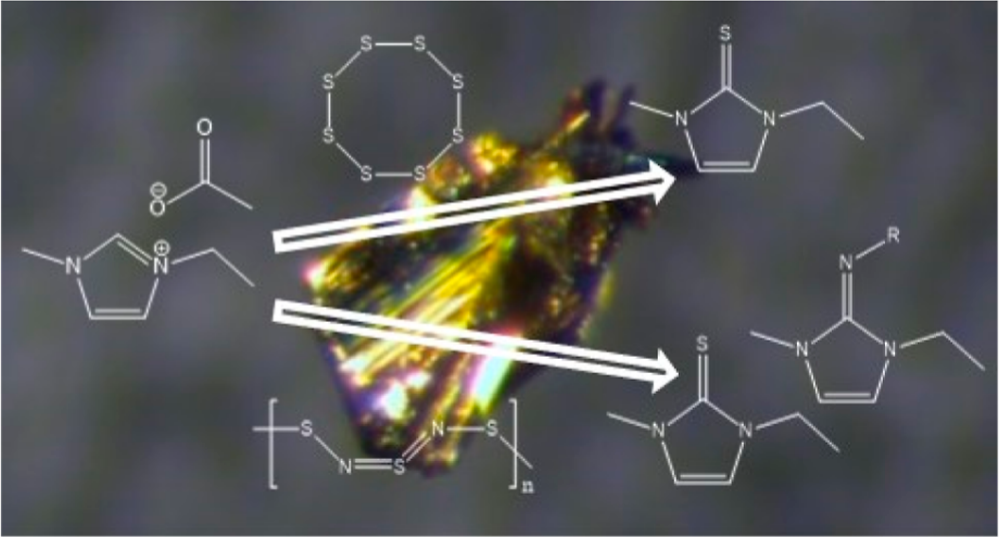

We investigate the reactive dissolution process of poly(sulfur nitride) (SN)_*x*_ in the ionic liquid (IL) 1-ethyl-3-methylimidazolium acetate [EMIm][OAc] in comparison to the process of elemental sulfur in the same IL. It has been known from the literature that during the reaction of S_8_ with [EMIm][OAc], the respective thione is formed via a radical mechanism. Here, we present new results on the kinetics of the formation of the respective imidazole thione (EMImS) via the hexasulfur dianion [S_6_]^2–^ and the trisulfur radical anion [S_3_]^•–^. We can show that [S_6_]^2–^ is formed first, which dissociates then to [S_3_]^•–^. Also, long-term stable radicals occur, which are necessary side products provided in a reaction scheme. During the reaction of [EMIm][OAc] with (SN)_*x*_ chains, two further products can be identified, one of which is the corresponding imine. The reactions are followed by time-resolved NMR spectroscopic methods that showed the corresponding product distributions and allowed the assignment of the individual signals. In addition, continuous-wave (CW) EPR and UV/vis spectroscopic measurements show the course of the reactions. Another significant difference in both reactions is the formation of a long-term stable radical in the sulfur–IL system, which remains active over 35 days, while for the (SN)_*x*_–IL system, we can determine a radical species only with the spin trap 5,5-dimethyl-1-pyrrolin-*N*-oxide, which indicates the existence of short-living radicals. Since the molecular dynamics are restricted based on the EPR spectra, these radicals must be large.

## 1 Introduction

Some ionic liquids (ILs) have a tremendous range of applications as green solvents caused by their special properties such as catalytic activity, negligible vapor pressure, and good water solubility.^1−9^ Compared to standard solvents, ILs are salts that can be liquid even at room temperature.^10−12^ 1-Ethyl-3-methylimidazolium acetate [EMIm][OAc] is one of the most frequently employed ILs considering its low melting temperature of −20 °C^13^ and its ability to break hydrogen bonds, which leads to the solubility of some important natural polymers, such as, e.g., cellulose or proteins.^14−17^ [EMIm][OAc] belongs to the group of N-heterocyclic ILs which can form reactive N-heterocyclic carbenes (NHCs) and acetic acid as a side product.^18−22^ With the formation of NHCs, a subtle balance between a physical dissolution process and a chemical reaction between solvent and solute occurs.^22−24^ The NHC can, e.g., react with certain polymers via a nucleophilic attack, whereby the polymer chains may degrade or be unintentionally modified.^25^ To prevent carbene formation, the pH of the system can be changed or other anions and cations or combinations of both can be selected for the ILs.^23−26^

**Figure 1:**
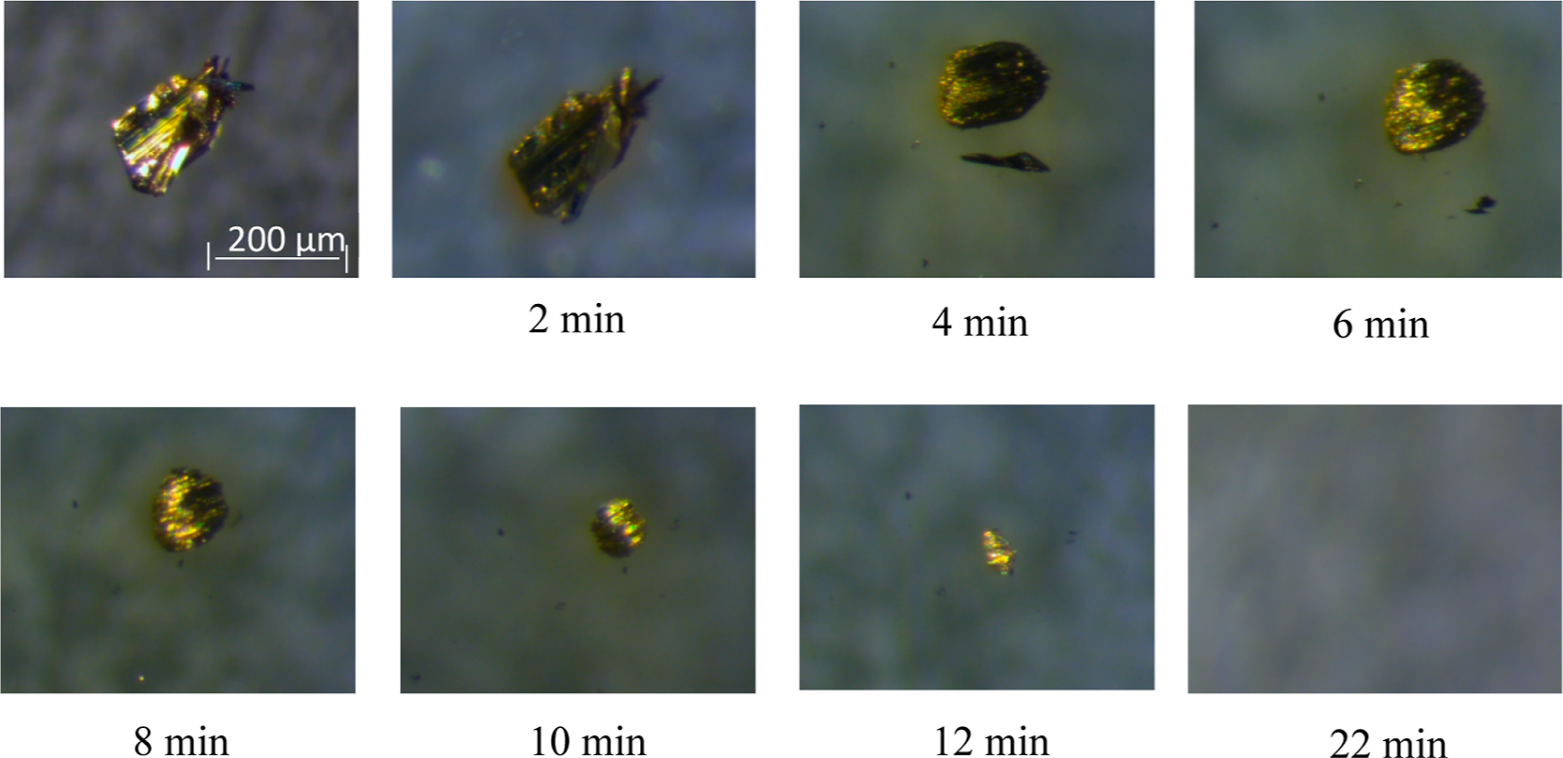
Dissolution process of (SN)_*x*_ in [EMIm][OAc] at 60 °C observed by polarized optical stereo microscopy (POSM) as a function of time.

The reactive dissolution of elemental sulfur in ILs^27−29^ and especially in [EMIm][OAc]^30^ is known, although many details of the reaction process are still under discussion. Boros et al.^27^ were able to show that [S_6_]^2–^ exists in the dissolution process of sulfur in an equilibrium with [S_3_]^•–^.^31−34^ NMR studies showed that the carbene of [EMIm][OAc] reacts with sulfur to produce the respective thione.^27,30,35^ With this background, sulfur shows an interesting chemistry with a plethora of high-tech applications.^36−39^ In combination with nitrogen, a whole range of sulfur–nitrogen compounds can also be produced, which offer also interesting application possibilities.^40−43^ One of these compounds is poly(sulfur nitride) (SN)_*x*_.^44−46^ The standard procedure for (SN)_*x*_ synthesis starts with the sublimation of heterocyclic S_4_N_4_ at elevated temperatures and high vacuum over silver wool to form the four-membered ring S_2_N_2_.^44,47,48^ Then, a topochemical solid-state polymerization of S_2_N_2_ crystals by ring opening to form (SN)_*x*_ occurs over several weeks at room temperature.^44^ This simple, high-crystalline inorganic polymer is interesting for its metallic conductivity of 1 to 4 × 10^3^ S·cm^–1^ at room temperature^48^ and its superconductivity below 0.3 K.^45^ (SN)_*x*_ is not soluble in any conventional solvent.^49^ This is a major disadvantage for the further characterization and modification of (SN)_*x*_. A comprehensive review on the synthesis and properties of (SN)_*x*_ is given by Banister and Gorrell.^50^ It has been known that (SN)_*x*_ can be converted with elemental bromine, which increases the conductivity by 1 order of magnitude.^45,51^ In general, it is considered that (SN)_*x*_ degrades over larger timescales in aqueous solutions or in air with high humidity.^49^ Fluck showed that diphenyldiazomethane can react with the binary S–N system S_4_N_4_ under the formation of nitrogen. He postulated that the attack of the nucleophile takes place primarily on the sulfur atom.^49,52^Figure 1 shows that, surprisingly, [EMIm][OAc] is suitable to dissolve (SN)_*x*_. It remains an open question if this is also a reactive dissolution process where the carbene of the IL is involved.

In this work, the reactive dissolution process of elemental sulfur (S_8_) in [EMIm][OAc] is investigated and compared with the process of dissolving (SN)_*x*_ in [EMIm][OAc]. ^1^H and ^13^C NMR spectroscopy is employed to study details of the processes for both systems and their kinetics, with the special focus on understanding whether (SN)_*x*_ is physically dissolved or chemical reactions are involved. Since the reaction mechanisms include radical species, we used time-dependent continuous-wave (CW) EPR spectroscopy measurements for their detection. Since several intermediate species were colored, additional UV/vis spectroscopic measurements were employed to study details of the occurrence of these species during the reaction/dissolution processes.

## 2 Experimental Section

### 2.1 Materials

[EMIm][OAc] (abcr, 98.0%), dimethyl sulfoxide-*d*_6_ (DMSO-*d*_6_, abcr, 99.8 atom % D), _S8_ (VWR Chemicals 99.9%), toluene (Carl Roth, ≥ 99.5%), ethanol (Carl Roth, ROTIPURA ≥ 99.8%), activated carbon (Merck), silica gel 60 (Carl Roth, 0.03–0.2 mm), 5,5-dimethyl-1-pyrrolin-*N*-oxide (DMPO), and methanol (Carl Roth, ROTISOLV HPLC Ultra Gradient grade) were used as received. The synthesis and characterization of (SN)_*x*_ crystals are described elsewhere.^44,46^

### 2.2 Reaction Procedure of S_8_ and (SN)_*x*_ in [EMIm][OAc]

S_8_ (32.2 mg, 0.125 mmol) was weighed in a vial with a stirrer under a nitrogen atmosphere and mixed with [EMIm][OAc] (600.7 mg; 3.529 mmol). Afterward, the vial was tightly closed with an aluminum cap. The reaction solution was stirred for 48 h at *T* = 60 °C. Then, the solution was purified by column chromatography with silica gel and a toluene/ethanol mixture (9:1). The obtained thione was isolated as a yellowish oily liquid (yield: 16.4 mg).

^1^H NMR (500 MHz, DMSO-*d*_6_, 27 °C): δ 7.14 (*d*, ^3^*J* = 2.15 Hz, 1H, HS–4), 7.11 (*d*, ^3^*J* = 2.15 Hz 1H, HS–5), 3.95 (*q*, ^3^*J* = 7.20 Hz, 2H, HS–7), 3.46 (*s*, 3H, HS–6), 1.21 (*t*, ^3^*J* = 7.19 Hz, 3H, HS–8) ppm; ^13^C NMR (126 MHz, CDCl_3_, 27 °C): δ 160.9 (CS–2), 118.3 (CS–5), 116.4 (CS–4), 41.9 (CS–7), 34.2 (CS–6), 14.0 (CS–8) ppm.

(SN)_*x*_ (28.3 mg; 0.307 mmol) was weighed in a vial with a stirrer under a nitrogen atmosphere, mixed with [EMIm][OAc] (1113.2 mg; 6.651 mmol), and finally, the vial was tightly closed with an aluminum cap. The reaction solution was stirred for 48 h at *T* = 60 °C. Then, the reaction solution was cleaned via column chromatography with silica gel and a toluene/ethanol mixture (9:1). The obtained thione and other products were isolated as a brown red oily liquid (yield: 65.7 mg). ^1^H NMR: (500 MHz, DMSO-*d*_6_, 27 °C): δ 7.14 (*d*, ^3^*J* = 2.34 Hz, 1H, HS–4), 7.11 (*d*, ^3^*J* = 2.34 Hz, 1H, HS–5), 6.48 (*d*, ^3^*J* = 2.94 Hz, 1H, HN–4), 6.43 (*d*, ^3^*J* = 2.80 Hz, 1H, HN–5), 3.95 (*q*, ^3^*J* = 7.23 Hz, 2H, HS–7), 3.50 (*q*, ^3^*J* = 7.26 Hz, 2H, HN–7), 3.46 (*s*, 3H, HS–6), 3.09 (*s*, 3H, HN–6), 1.22 (*t*, ^3^*J* = 7.15 Hz, 3H, HS–8), 1.13 (*t*, ^3^*J* = 7.23 Hz, 3H, HN–8) ppm. ^13^C NMR (126 MHz, DMSO-*d*_6_, 27 °C): δ 160.9 (CS–2), 118.3 (CS–5), 116.4 (CS–4), 111.3 (CN–5), 109.5 (CN–4), 41.9 (CS–7), 37.5 (CN–7), 34.2 (CS–6), 29.7 (CN–6), 14.6 (CN–8), 14.0 (CS–8) ppm.

### 2.3 Characterization

#### 2.3.1 Nuclear Magnetic Resonance Spectroscopy

^1^H and ^13^C NMR spectroscopic measurements were performed using an Agilent Technologies 500 MHz DD2 spectrometer at *T* = 27 °C. Coaxial inserts for NMR sample tubes were used to examine the samples in [EMIm][OAc]. The sample of interest was transferred to the inner sample tube under a nitrogen atmosphere, and the outer tube was filled with DMSO-*d*_6_. The evaluation of the NMR spectroscopic data was carried out using MestReNova version 9.0. All NMR spectra were calibrated to DMSO-*d*_6_, and the corresponding signals were assigned and integrated.

For the preparation of a stock solution, S_8_ (35.5 mg, 1.107 mmol) and [EMIm][OAc] (718.9 mg, 4.223 mmol) were added to a glass vial at *T* = 60 °C and stirred for 24 h under a nitrogen atmosphere. A stock solution of (SN)_*x*_ (37.2 mg, 0.202 mmol) in [EMIm][OAc] (1204.3 mg, 7.075 mmol) was also prepared by the same procedure. The stock solutions were then diluted with different amounts of IL (Supporting Information Tables S1 and S2).

#### 2.3.2 Electron Paramagnetic Resonance Spectroscopy

Room-temperature CW EPR measurements at X-band frequency (∼9.4 GHz) were performed on a Magnettech MiniScope MS400 benchtop spectrometer (Magnettech, Berlin, Germany; now Bruker BioSpin, Rheinstetten, Germany). EPR spectra were recorded with a microwave power of 10 mW, 100 kHz modulation frequency, modulation amplitude of 0.2 mT, and 4096 points. Each of the shown spectra was accumulations of six scans; each took 30 s. The low-temperature spectra were recorded with the same parameters at liquid nitrogen temperature. The EasySpin software package (version 6.0.0-dev.51) was used for the simulations of the EPR spectra. We considered natural abundance of nuclei through simulations, if not mentioned otherwise.^53^

A suspension of 1 wt % elemental sulfur (2.6 mg, 0.081 mmol) in [EMIm][OAc] (199.6 mg, 1.173 mmol) was prepared under a nitrogen atmosphere and then immediately taken up with a capillary so that some S_8_ crystals with IL were transferred into the capillary. Finally, the capillary was sealed with Critoseal at both ends. There was a maximum time of about 10 min between sample preparation and measurement.

By EPR spin trapping experiments, a suspension of (SN)_*x*_ in the IL was prepared as follows. The spin trap DMPO (10.5 mg, 0.093 mmol) and [EMIm][OAc] (407.9 mg, 2.396 mmol) were mixed, and activated carbon (17.7 mg) was added under a nitrogen atmosphere. Activated carbon was added to remove any possible oxidation products of DMPO. The resulting solution was subsequently filtered off from the activated carbon. Then, the filtered solution was mixed with 2 mg of (SN)_*x*_ crystals, filled into a capillary immediately, and sealed.

#### 2.3.3 UV/Vis Spectroscopy

All UV/vis-absorption measurements were performed on a PerkinElmer LAMBDA 365 UV/vis spectrophotometer using Hellma Analytics quartz glass cuvettes (*d* = 10 mm). Temperature control was achieved using a PerkinElmer Peltier System L365. The samples were weighed [1 mg of S_8_ or (SN)_*x*_, 100 μL, 2 mL of DMSO] and sealed in a nitrogen atmosphere. First, a blank spectrum of [EMIm][OAc] and DMSO was recorded and set as the baseline. The spectra of the samples were recorded every 10 min; for S_8_, additionally at the beginning, measurements were taken in steps of 1 min. The measurements were recorded at *T* = 20 °C between 300 and 700 nm. The data evaluation was performed using OriginPro 2019.

#### 2.3.4 Polarized Optical Stereo Microscopy

Polarized optical stereo microscopy (POSM) was performed on a Discovery.V8 SteREO instrument from Carl-Zeiss-Jena (Germany). The attached digital camera was an Axiocam ERc5s.

## 3 Results

### 3.1 Sulfur in [EMIm][OAc]

#### 3.1.1 NMR Characterization

**Figure 2:**
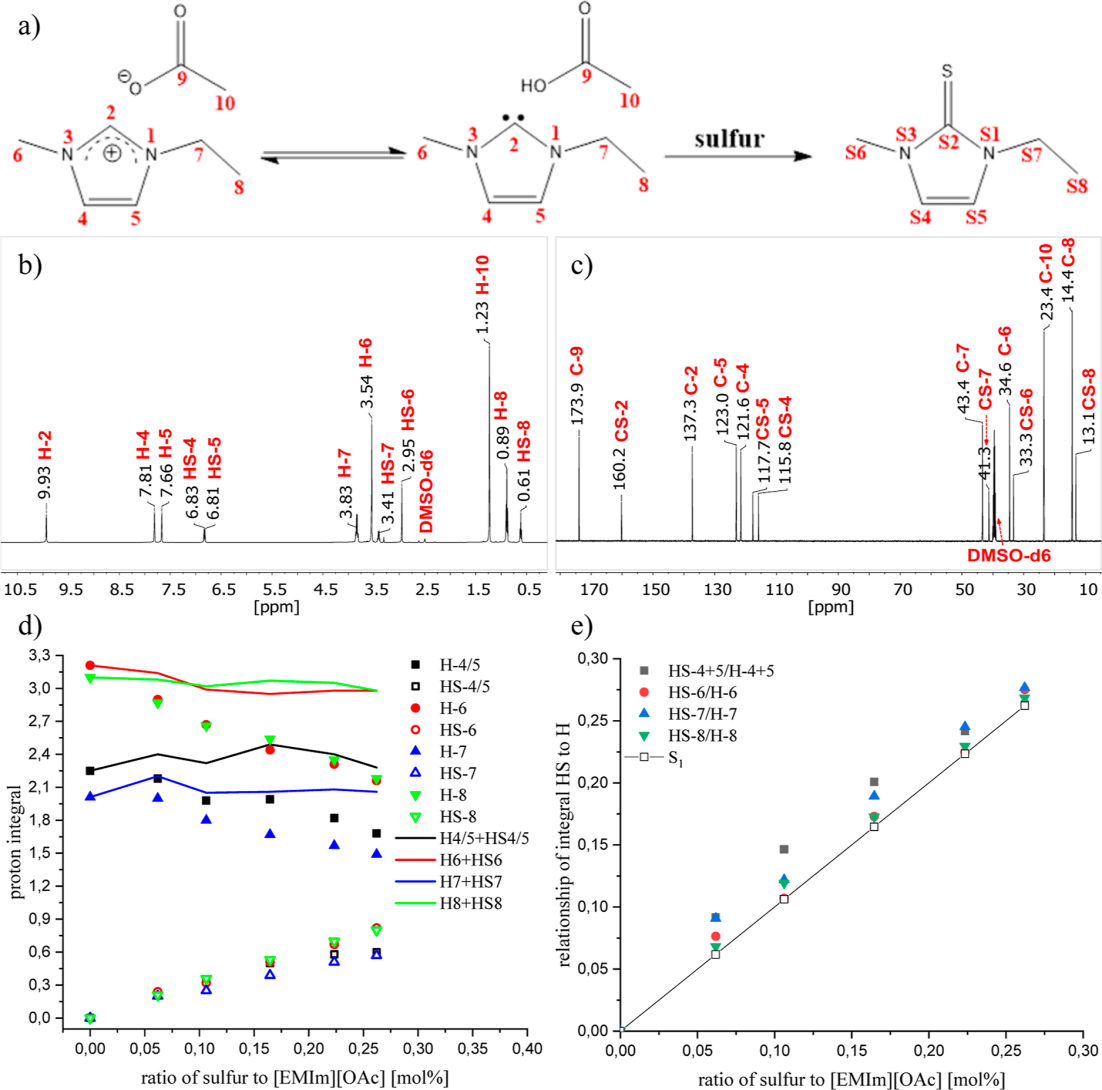
(a) Equilibrium between [EMIm][OAc] and the respective carbene, which can react with S_8_ to form the thione EMImS. ^1^H (b) and ^13^C NMR (c) spectra of 5 wt % S_8_ after reactive dissolution in [EMIm][OAc] at room temperature. (d) Integral of proton signals as a function of the molar ratio of S to IL. (e) Proton intensity ratios of the HS-X signal to the H-*X* signal. The theoretical black line indicates the exact attachment of one sulfur atom to an IL molecule.

As discussed above, it has been known that [EMIm][OAc] is a reactive solvent for elemental sulfur.^27^ The IL [EMIm][OAc] is in equilibrium with the respective carbene and the corresponding acetic acid.^18−22^ This carbene can react with S_8_ under the formation of 1-ethyl-3-methylimidazole-2-thione (EMImS) (Figure 2a).^27^ The ^1^H NMR spectrum of S_8_ after the reactive dissolution process in [EMIm][OAc] (Figure 2b) shows the characteristic signals of the IL. The signals are assigned as H-*X*, where *X* represents the respective position of the proton (Figure 2a). For example, the proton at position H-2 can be found as a singlet at a chemical shift of 9.93 ppm. In addition to the proton signals of [EMIm][OAc], new signals can be found as a result of the chemical reaction of S_8_ with the carbene. They are indicated as HS-*X*. The new signals appear next to those of the IL. Thus, two doublets can be detected at 6.83 ppm (HS-4) and 6.81 ppm (HS-5). Since EMImS does not have a proton in the H-2 position (C=S bond), it is reasonable that no new peak occurs next to this position, as shown in Figure 2a. The ^13^C NMR spectrum (Figure 2c) shows more characteristic signals of [EMIm][OAc] and EMImS.^30,54^ In the ^13^C NMR spectrum of the reaction product, the carbon signal for position 2 can be found at 160 ppm (Figure 2c). This is characteristic for EMImS.^55^ The NMR spectra of purified EMImS are shown in Supporting Information Figure S7.

From concentration-dependent ^1^H NMR measurements, the integrals of the signals were determined (Supporting Information Tables S3 and S4), and a manual calibration was performed by setting the proton signal from the methyl group of the acetate ion (H-10) to 3.00 (Supporting Information Figures S1–S6). A decrease in the proton signals of the IL and an increase in the proton signals of the product were observed (Figure 2d). The sum of the integrals of the signals of H-*X* and H-SX must be identical with the integral of H-*X* at time zero. This holds true for all integrals (full lines in Figure 2d), except for position 2 of the imidazole ring, where the proton is replaced by a sulfur atom of the respective thione.

The assumed thione formation is confirmed again by the plot of the integral ratios (Supporting Information Table S6) of HS-*X* to H-*X* as a function of the sulfur content in the solution (Figure 2e). If the ratios determined from the spectra are compared with the molar ratios of a sulfur atom to the IL as 1:1 (black line), the values are quite close. This is an indication that only one sulfur atom is attached to the imidazole ring, and all sulfur atoms are converted. The fact that the determined ratios from the spectra deviate slightly from the black line can be explained by the manual calibration, which was set to 3.00 for methyl protons (H-10), and all integrals were scaled according to that.

#### 3.1.2 UV/Vis Characterization

**Figure 3:**
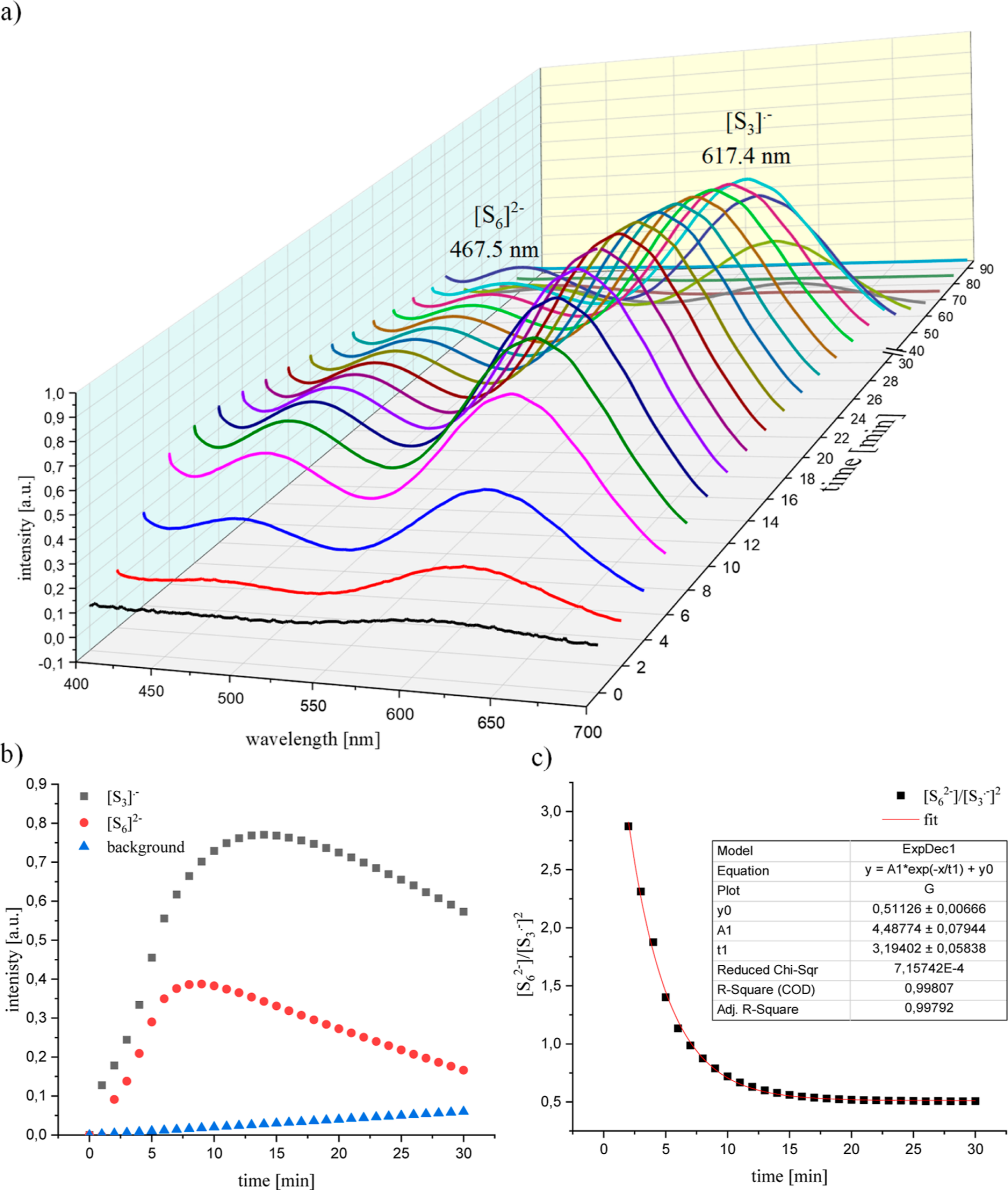
(a) Time-dependent UV/vis spectra from 0 to 90 min at room temperature of the dissolution process of S_8_ (1 mg) in a mixture of IL (0.1 mL) and DMSO (2.0 mL). (b) Intensity of the [S_6_]^2–^ and [S_3_]^•–^ signals as well as the background signal. (c) [S_6_]^2–^/[S_3_]^•–^ ratio as a function of time.

The UV/vis spectra (Supporting Information Figure S15) give an indication of which intermediates are formed during the reaction between S_8_ and [EMIm][OAc]. As the [EMIm][OAc] itself shows a strong absorption at 350 nm, the measurements were performed in a mixture of IL and DMSO. After S_8_ was added to the DMSO-IL mixture, two distinct bands appeared at 467 and 617 nm, respectively (Figure 3a). The band at 467 nm shows a maximum after 8 min reaction time, while the band at 617 nm increases further and has an intensity maximum after 14 min before it decreases (Figure 3b).

According to Boros et al., the peak at 617 nm is caused by a trisulfide radical anion [S_3_]^•–^, which has a characteristic blue color,^27,32,34,56−59^ also observed during the reaction. The [S_6_]^2–^ dianion has an absorption band of 467 nm. Corresponding to Steudel and Chivers, both poly sulfur compounds are in equilibrium according to formula 1.^32,60,61^
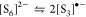
1

The full kinetics are described in Supporting Information Formulae S1–S10 and Figures S16 and S17. The measured UV-intensities are taken as concentration proportional quantities (Figure 3c). The value for [S_6_]^2–^ is corrected by a linear increasing background due to the broadening of the IL signal (Figure 3b). Obviously, the reaction runs first through a high extent of [S_6_]^2–^, before it approaches a constant value after an exponential decay  with *k*_eq_ = 0.313 min^–1^ (Figure 3c).

#### 3.1.3 EPR Characterization

**Figure 4:**
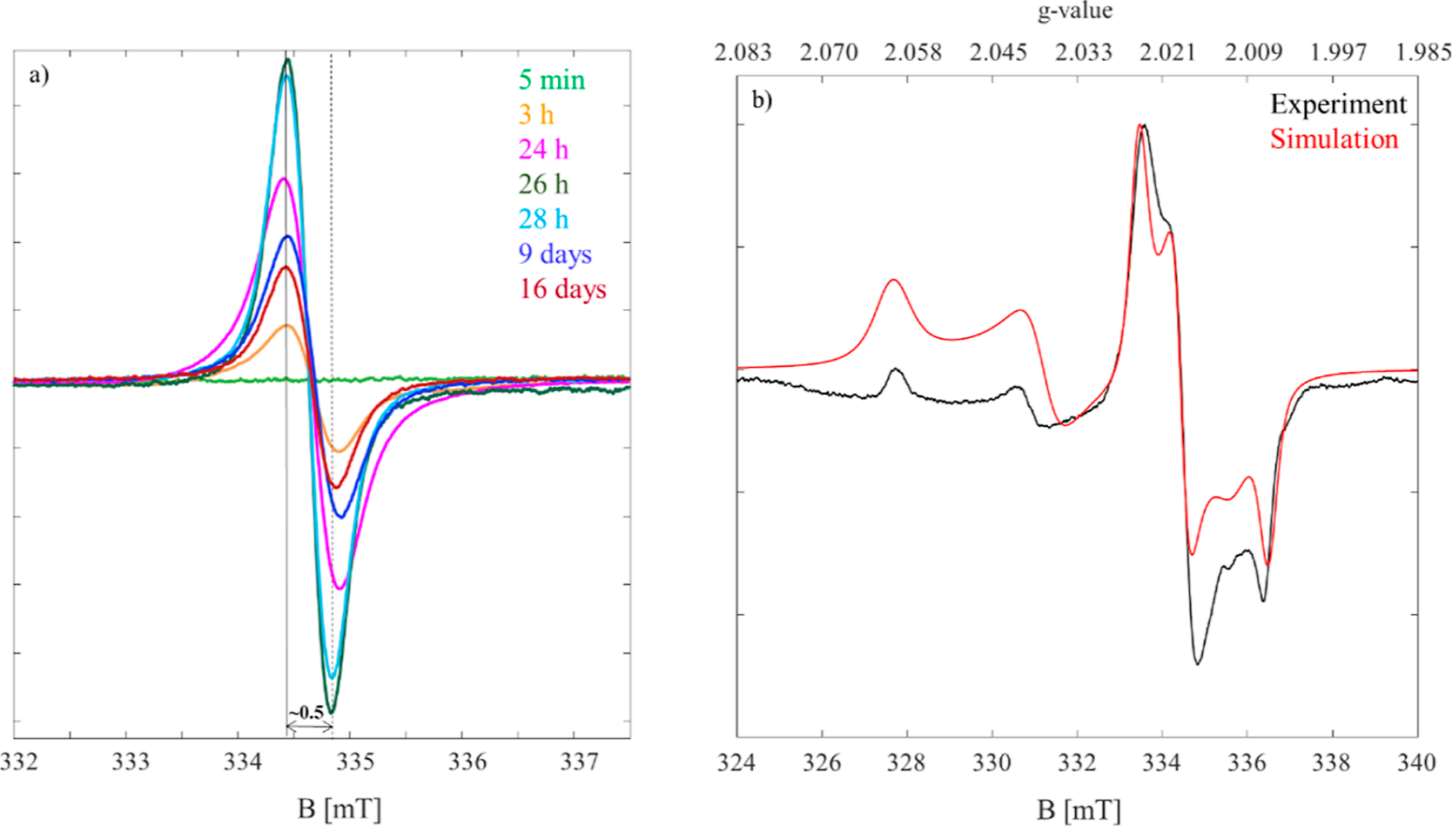
(a) Room-temperature EPR spectra of S_8_ in [EMIm][OAc] evolving from 0 h to 16 days. For clarity, we have shown a selection of measured spectra. The peak to peak line width is about 0.5 mT. (b) Experimental low-temperature (77 K, black) and simulated (red) EPR spectra of S_8_ in [EMIm][OAc]. Corresponding *g*-values are given in the upper *x*-axis.

To analyze the formation of radical reaction products between S_8_ and the IL, we conducted time-dependent EPR measurements (Figure 4a). We started the first measurement 10 min after sample preparation at room temperature. The signal intensity increased with time until 26 h where the highest intensity was observed. After 26 h, the sample was heated to 60 °C for 2 h to test whether a temperature increase influences the stability of the radical (Supporting Information Table S8). Accordingly, an EPR measurement was performed after 28 h, which showed that only a slight decrease in the intensity can be observed. It can hence be concluded that the temperature increase has no significant influence on the radical stability.

Then, the sample was cooled to room temperature again. After 9 days, the signal could still be observed clearly, although with smaller intensity. Even after 16 days, we were still able to detect the signal. These observations are indicative of a persistent radical formed during the reaction between S_8_ and IL. A measurement taken after 35 days showed the same signal (Supporting Information, Figure S18).

Although all measured EPR spectra had a constant peak-to-peak distance of ∼0.5 mT, we observed a line broadening with time. This could be an indication of the presence of several radical species with unresolved hyperfine couplings.

The room-temperature EPR spectra are characterized by a *g*_iso_ of 2.012. Such a rather strong deviation from *g*_e_ ∼2.0023 to higher values is a sign of an inorganic radical, a sulfur-based radical in this case. A comparison of the obtained *g*-values with those reported in the literature^62^ corroborate the interpretation of the presence of a sulfur-based radical. The *g*-value of sulfur in 1,3-diisopropenyl benzene can be determined at 2.0044^63^ or in NaOtBu and DMF at 2.0292.^57^ However, we cautiously attribute this signal to the trisulfide radical anion [S_3_]^•–^. Surveying the literature about [S_3_]^•–^, we found that this radical is characterized in two different forms: a common form, which is an isotropic signal with *g*_iso_ ∼2.029,^64,65^ and a rhombic signal that is observed in different materials.^31,62,66−69^ The reported *g*-values are dependent on the local environment of the radical. We also found a temperature-dependent behavior of this radical in the literature, which completely matches our observed EPR spectral series (Figure 4a,b). Low-temperature EPR measurements at 77 K revealed a highly anisotropic signal (Figure 4b), characteristic of trisulfide anion radicals, mostly investigated for ultramarine (UM) pigment samples. The electron spin echo of a trisulfide radical in blue UM at 1.4 K^66^ shows a clearly rhombic *g*-tensor (2.046, 2.036, 2.005). Similar values were reported by Baranowski et al. (2.050, 2.033, and 2.004) measuring trisulfide radical centers of UMs.^70^ Goslar and co-workers reported g-values (2.016, 2.033, and 2.050) for temperature ranges between 4.2 and 50 K for [S_3_]^•–^.^69^ Raulin et al.^71^ reported *g*-values (2.054, 2.041, and 2.005) at 30 K for [S_3_]^•–^ of different UM pigments. DFT studies on UMs also returned a rhombic g-tensor (2.002, 2.056, 2.040) for trisulfide radical.^72^

Spectral simulations confirmed that the EPR spectrum is consistent with the assumption of two radical species with hyperfine coupling to a nuclear spin *I* = 3/2 and is dominated by *g*-anisotropy. The first contribution to the spectrum (∼25% of the whole spectrum) is a sulfur species with *g*-values (2.060, 2.038, and 2.015), which contributes to the higher field part of the spectrum. The second sulfur radical contributed to the lower field of the spectrum, indicating *g* values (2.020, 2.014, and 2.001). The first component (high field) resembles the reported [S_3_]^•–^, both in terms of spectral shape and *g*-value. Therefore, we attribute the observed signal to two different species: a combination of trisulfide radicals and a new sulfur-based radical. The presence of two radical components with different dynamics (mobilities) could also explain the broad line width of observed EPR spectra at room temperature. Since there is no crystal structure available for the trisulfide radical, theoretical studies suggest a *C*_2*v*_ symmetry.^72,73^ Comparing the obtained results in this study with those in the literature, one might conclude that the symmetry of the trisulfide radical in the IL is preserved.

EPR measurements of pure [EMIm][OAc] did not result in an EPR signal. Therefore, we can discard the possibility that the observed EPR signal originates solely from the IL or the carbene (Supporting Information Figure S19a). The imidazole carbene is in a singlet state, which means that both electrons are paired in one orbital^74−76^ and does not give an EPR signal. Accordingly, the carbene, as a nucleophile, can break the S_8_ ring and start the reaction. This also means that the resulting EPR signal must originate from sulfur-containing molecular species. The possible origins for the generation of such sulfur-based radicals are discussed in detail in the Discussion section.

#### 3.1.4 Discussion

From the NMR data, we can conclude that the final product in the reaction of S_8_ with [EMIm][OAc] is 1-ethyl-3-methylimidazole thione EMImS. During reaction, the polysulfide compounds [S_6_]^2–^ and [S_3_]^•–^ are formed, beginning with an excess of [S_6_]^2–^. Following this, we see a decrease in the absorption intensities over time, which indicates that [S_3_]^•–^ is degraded by the carbene. EPR characterization shows that the reaction produces a long stable radical species with a characteristic *g*-value of sulfur compounds. Low-temperature (77 K) measurements reveal a highly rhombic structure, in contrast to the EPR signal measured at room temperature. Analyzing the spectrum more deeply by spectral simulations further showed the presence of two radical species in the S_8_–IL system. For this, [S_3_]^•–^ (about 75% of spectral contribution) and a long-term stable radical species, perhaps a thione radical, were detected.

**Scheme 1:**
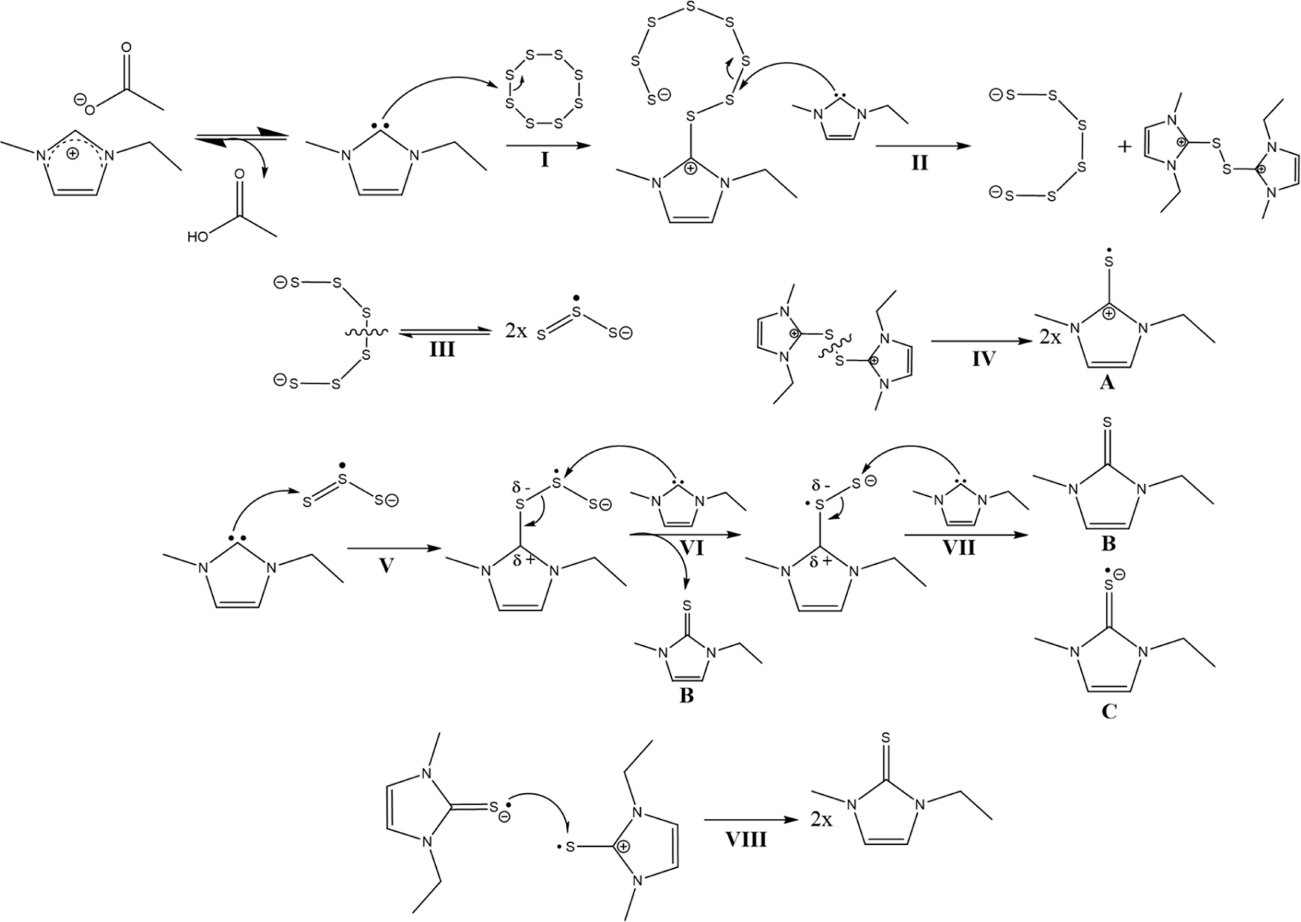
Postulated Reaction Mechanism of S_8_ with [EMIm][OAc]

In summary, the reaction of S_8_ with [EMIm][OAc] appears more complex than anticipated, and a variety of intermediates and end products are formed. In Scheme 1, we postulated a possible reaction mechanism of S_8_ with the IL based on our experimental data. For the initial degradation of S_8_, Tarasova et al.^29^ propose two possibilities. Either the S_8_ is gradually degraded by nucleophilic attack, or the S_8_-ring is broken and splits into two [S_4_] chains, which can then be further degraded. In contrast, Sharma and Champagne^28^ propose a mechanism in which the ring is broken by an attack of a nucleophile (Nu) with a second Nu; (NuS)_2_ and [S_6_]^2–^ are formed by a second attack of a nucleophile on the second sulfur atom in NuS_8_. UV/vis measurements show a fast formation of [S_6_]^2–^ in excess against [S_3_]^•–^, so the process via [S_4_] can be excluded. Between S_8_ and [S_6_]^2–^, no intermediates could be detected, so we are ultimately unable to say exactly what mechanism is used to break down the S_8_ chain; we simplified the scheme to [S_8_] → [NuS_8_] → [S_6_] + (NuS)_2_.

First, to form the reactive carbene on the imidazole ring, the proton at position 2 must be abstracted from the acetate counterion. Once the carbene is formed, it can attack a sulfur atom on the S_8_ ring (**I**) and form an intermediate. This intermediate compound can attack the second sulfur (the one adjacent to the sulfur bound to the imidazole) and forms the corresponding [S_6_]^2–^ and a disulfur intermediate (**II**). Step **IV** could break the S–S bond and generate two radical cationic structures **A**, and after step **III**, the [S_6_]^2–^ would be in equilibrium with the corresponding [S_3_]^•–^. Accordingly, the formed [S_3_]^•–^ would be attacked by a carbene after step **V**, whereupon the next carbene would attack the middle sulfur after step **VI** and form the corresponding EMImS **B**.^77^ However, at this point, it cannot be completely clarified what happens further, but we present here one possible reaction pathway of the sulfur–IL system. Other reaction mechanisms are shown in the Supporting Information Chapter 4.

It can be assumed that under preservation of the electron configuration, the sulfur atom with the negative charge is attacked by a carbene (**VII**). This would produce EMImS **B** and radical anionic sulfur species **C**. The characteristic band of [S_2_]^−^ could not be detected in UV/vis 390 nm as it overlaps with the IL signal. In NMR data, no proton in the 2 position is found when sulfur was attached, so species **C** will not carry an additional proton but may transfer an electron to species **A** and form 2 thiones (**VIII**).

### 3.2 (SN)_*x*_ in [EMIm][OAc]

#### 3.2.1 NMR Characterization

**Figure 5:**
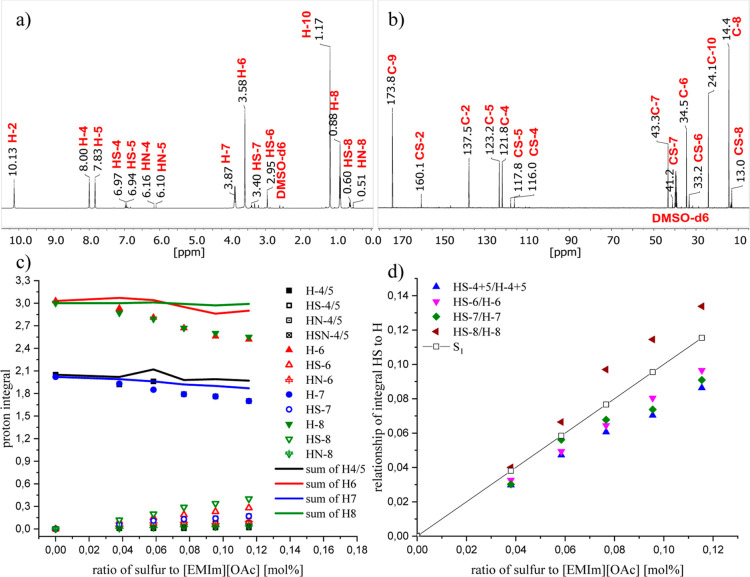
(a,b) ^1^H and ^13^C NMR spectra of 3 wt % (SN)_*x*_ after reactive dissolution in [EMIm][OAc] at room temperature. (c) Integral of proton signals as a function of the molar ratio of (SN)_*x*_ to IL. (d) Proton intensity ratios of the HS-*X* signal to the H-*X* signal. The black line indicates the exact attachment of one sulfur atom to one IL molecule.

The ^1^H NMR spectrum (Figure 5a) indicates a complex reaction between (SN)_*x*_ and [EMIm][OAc]. Proton signals at 6.97 ppm (HS-4) and 6.94 ppm (HS-5) can be observed, indicating the formation of EMImS. Consistently, the ^13^C NMR spectrum shows the characteristic signal of CS-2 at 160.1 ppm (Figure 5b). Additionally, the NMR spectra show all characteristic signals for the IL and the formed EMImS.^30,55^

Remarkably and in addition to the respective thione, further signals can be found in the ^1^H and ^13^C NMR spectra, suggesting a broader product distribution for the reaction of (SN)_*x*_ with [EMIm][OAc] compared to the S_8_–IL system. At 6.16 and 6.11 ppm, additional signals can be found that belong to a product that could possibly carry a nitrogen atom. Since nitrogen is present in (SN)_*x*_, the formation of an imine cannot be excluded. Another indication of a rather broad product distribution of (SN)_*x*_ in [EMIm][OAc] is found in the region of the proton signals of the methyl group at position 8, where an additional triplet appears at 0.51 ppm. In addition to the product signals of EMImS, other signals can also be found in the ^13^C NMR spectrum. For example, a carbon signal can be detected at 151.7 ppm (Supporting Information Figure S14), which could match a carbon atom with a double bond to a nitrogen atom.^78^

In the next step, we performed experiments with different amounts of (SN)_*x*_ in [EMIm][OAc]. The NMR spectroscopic measurements (Supporting Information Figures S8–S12) had to be carried out with amounts of only up to 3.00 wt % of (SN)_*x*_ which is the solubility limit of (SN)_*x*_ in [EMIm][OAc] (Experimental 2.3.2). When the proton integrals (Supporting Information Table S4) are plotted against the content of the sulfur present in the IL, the integrals of the IL decrease with increasing (SN)_*x*_ concentration (open symbols in Figure 5c). Accordingly, the product signals increase in intensity (see the full symbols in Figure 5c). When summing up the protons of the IL with those of the products, a nearly constant value should appear (full lines in Figure 5c). Small deviations can also be explained here by the manual integration or by setting the integral of the methyl group to 3.00. Since the IL integral decrease correlates with the increase in product intensities, it appears that one imidazole ring at a time removes one sulfur atom from the polymer chain and forms EMImS. Furthermore, it could also abstract a nitrogen atom out of the polymer chain to form more products (Supporting Information Figure S13).

To confirm this assumption, the ratios of the proton integrals of the product to the IL (full symbols in Figure 5d) were then plotted against the molar ratios of sulfur in (SN)_*x*_ to the IL (Supporting Information Table S6). The full line represents the case of one sulfur atom being attached on one imidazole ring. This shows that not each sulfur atom reacts with an imidazole ring as previously observed but that the ratios determined from the spectra are below the molar ratios. This indicates that not all of the sulfur in (SN)_*x*_ is converted to the thione. According to the values, about 80 mol % must convert to the corresponding imidazole thione and the remaining 20 mol % to other products. This would also explain the broader product distribution in the NMR spectra.

**Figure 6:**
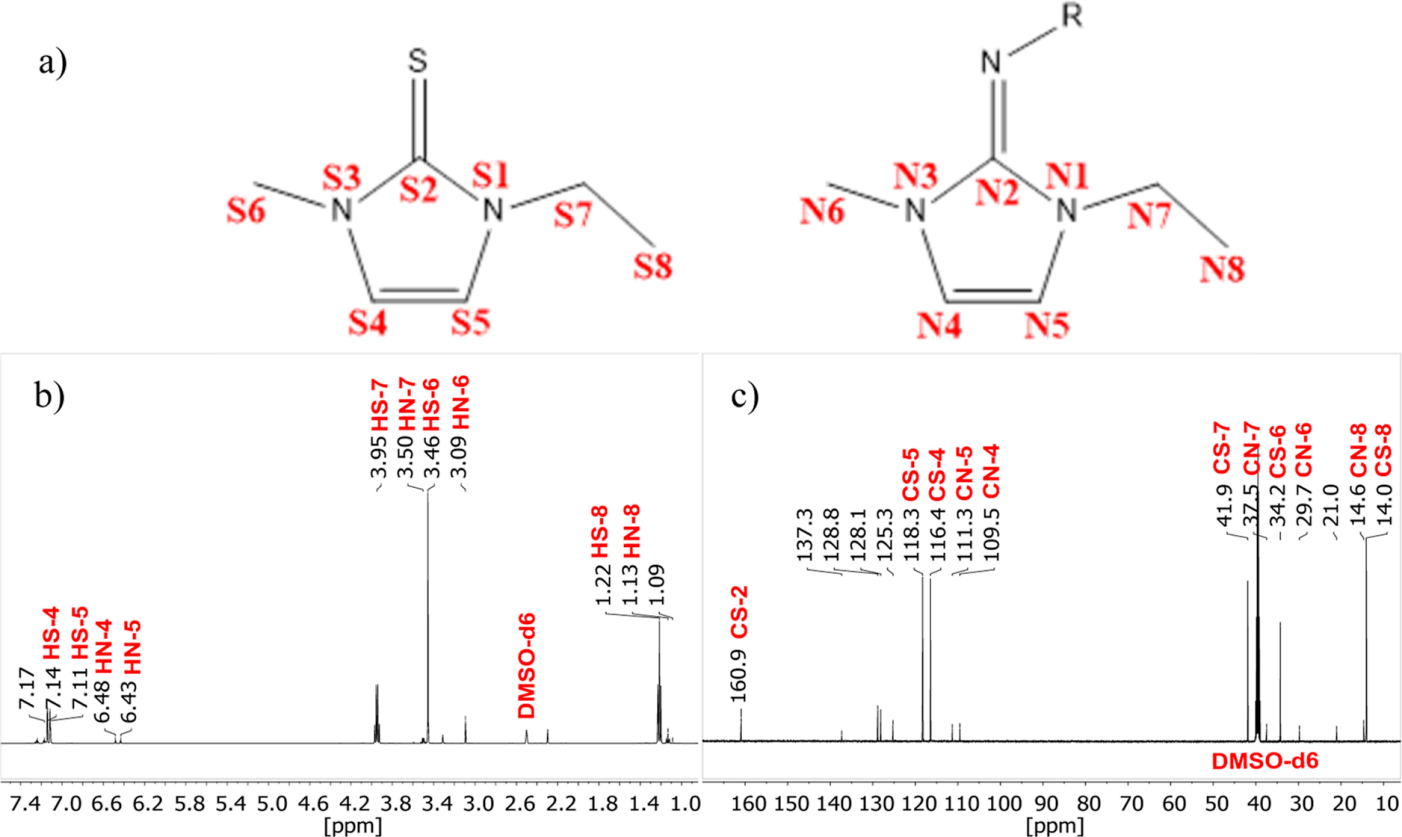
Chemical structure of the thione and imine formed by the reaction of (SN)_*x*_ with [EMIm][OAc] (a). ^1^H NMR (b) and the ^13^C NMR spectra (c) of the reaction products (R=H, S, S=N–, S–N=S and other possibilities of sulfur–nitrogen bonds).

^1^H and ^13^C NMR data after column chromatographic cleanup of the solution of (SN)_*x*_ in [EMIm][OAc] show all signals of the imidazole thione (Figure 6b,c).^55^

In addition to the formation of thione, other products could be identified in the spectra. However, it is not possible to exactly ascribe all of the substances to these spectral signatures. The spectra probably indicate the formation of several imidazole-based substances since the additional signals show the same splitting pattern as is usual for the protons in the imidazole ring. However, they do not give any information about the substitution residues at position 2. Since we were able to show that not all sulfur reacts to form the thione, it is possible that smaller polymer fragments are substituted on the carbon. Either a sulfur atom is still attached to the imidazole, which carries a chain of further nitrogen–sulfur residues, or a nitrogen with a polymer chain is attached to the imidazole ring and forms an imine compound as a product.

#### 3.2.2 EPR Characterization

Unlike the behavior of S_8_ in [EMIm][OAc], where a persistent radical is formed, (SN)_*x*_ shows a completely different reaction behavior in [EMIm][OAc]. No radical species could be detected over a period of 3 h (Supporting Information Figure S19b). There are two possibilities to explain this observation: (a) either no radical species is formed during the reaction in the case of the (SN)_*x*_ with the IL or (b) a radical is formed and has a lifetime too short to be detectable, hence serving as an intermediate. To examine the latter possibility, we used DMPO as the spin trap (Materials) to scavenge a short-lived radical and transform it into a persistent, EPR-detectable radical.

**Figure 7:**
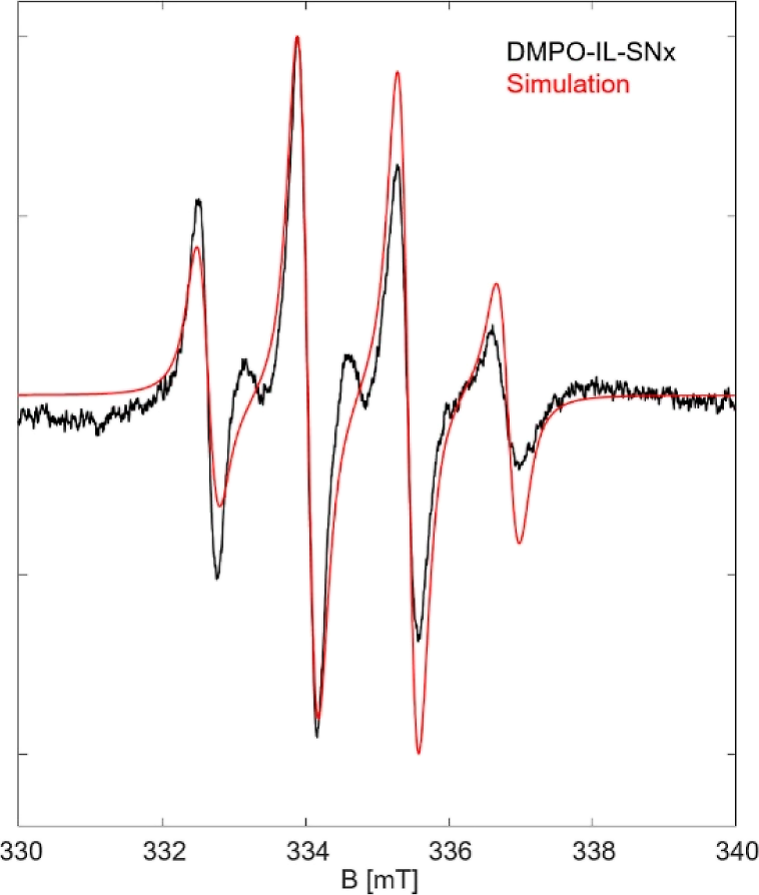
Room-temperature EPR spectrum of (SN)_*x*_ in [EMIm][OAc] and the DMPO spin trap (black). The corresponding simulations (red) revealed the presence of polymer strands nearby each other and polymer strands that contain radical centers.

The results of EPR spin trapping with IL-(SN)_*x*_ are shown in Figure 7. At first sight, one can observe that the reaction of (SN)_*x*_ with the IL produces a clearly different radical species (from that of the S_8_–IL reaction) that can be considered as a short-lived intermediate. Interestingly, we obtained a broad and damped high-field EPR signal of the spin trap, which is in contrast to usually observed sharp and narrow lines in DMPO spin trapped systems.^79−81^ The experimental spectrum could be well simulated with *g*_iso_ = 2.012 and hyperfine splittings of A (^14^N) = 1.41 mT and A (^1^H) = 1.36 mT. These hyperfine splitting parameters are similar to those known for DMPO spin adducts of sulfur-based radicals.^82^ To simulate the highly suppressed low field line, however, we needed to consider two species with the same coupling but different dynamics. The main species (about 75% contribution to the whole spectrum) features a highly restricted motion and an exchange coupling of about 8 MHz. The presence of exchange couplings can be explained due to high local concentrations of radicals residing in close proximity (∼1.3 nm or 13 Å). This means the radical that binds to DMPO carries a large molecular residue, which in turn restricts the rotational motion of the radical adduct and apparently is locally enriched in concentration. This also describes the heavily damped high field line of the EPR spectrum.^83^

These observations can be explained by carbene attacking the polymer chain, which subsequently results in the formation of radical polymer fragments. This in turn could react at the DMPO and, depending on the chain length, cause broadening of the EPR signal.^84^

#### 3.2.3 Discussion

Since we know that the carbene must be formed to break the S_8_ ring and allow the reaction to start, it is highly likely that this also happens in the reaction of (SN)_*x*_ with the IL. The NMR spectra show that the EMImS is indeed formed, as in the case of S_8_. However, not only the thione is formed but also other products can be found. Accordingly, the variety of reaction products is much greater than for S_8_. This also seems logical since we have alternately linked sulfur and nitrogen atoms in the (SN)_*x*_. It was also shown that not all sulfur reacts with an imidazole ring, but only a certain fraction. Presumably, individual sulfur atoms are abstracted from the polymer chain, leaving smaller polymer fragments that can react subsequently. It is assumed that imidazole imines are formed, which carry smaller polymer fragments.

The EPR measurements, however, show intermediates, short-lived radicals that could only be detected by adding a spin trap. This indicates that short-lived radical compounds are produced in the system and decay quickly. Furthermore, the EPR spectra show that the resulting compounds must be large molecules since the spectra show restricted dynamics. This could be an indication that the resulting polymer fragments carry radicals, which can then react further with the carbene and thus increase the product variety. This seems to be the most logical step according to the corresponding characterization methods.

## 4 Conclusions

Using the analytical methods employed here, we showed that both S_8_ and (SN)_*x*_ react with [EMIm][OAc] to form different products. For the S_8_–IL system, thione EMImS is formed as the final product, with [S_6_]^2–^ and [S_3_]^•–^ as intermediates. Time-dependent measurements enabled a kinetic observation of their equilibrium and also showed that sulfur was further degraded by the carbene. EPR measurements confirmed that in addition to the formation of EMImS, a long-term stable radical was also formed, which could still be detected after 35 days.

The characterization of the S_8_–IL system provides information for understanding the corresponding (SN)_*x*_–IL system. The presence of nitrogen in the polymer leads to a wider range of reactions, but NMR spectroscopy shows that the corresponding thione is still the dominating product, suggesting that the polymer chain is also degraded by the carbene form of the IL. Also, during the reaction of (SN)_*x*_ in the IL, intermediate compounds are formed, which are degraded in the further course. EPR measurements are possible only in the presence of spin traps (DMPO). The results of EPR spin trapping experiments of the (SN)_*x*_–IL system suggest the presence of sulfur-based intermediate radical compounds. Further investigations of the (SN)_*x*_–IL system are of great interest to allow for a more detailed characterization of the reaction mechanism. Further types of ILs will be considered, e.g., triphenylphosphines or triazolium salts.^11,28^

## Supporting Information Available

Weight and molar ratios of S_8_ and (SN)_*x*_ in [EMIm][OAc], NMR integrals of S_8_ and (SN)_*x*_ in the IL, chemical shifts of the solutions of S_8_ and (SN)_*x*_, respectively, in IL prior and after reaction with IL, expected and measured molar ratio of sulfur to IL and the NMR integral ratio of HS to IL, NMR characterization of S_8_ and (SN)_*x*_, respectively, in IL and the products of the reaction, UV/vis characterization data, additional EPR data of S_8_ and (SN)_*x*_ in IL, EPR measurement values of S_8_ as a function of time, and alternative reaction mechanisms of S_8_ in IL (PDF)

## Abbreviations

AC: activated coal
APT: attached proton test
DMF: dimethylformamide
DMPO: 5,5-dimethyl-1-pyrrolin-*N*-oxide
DMSO: dimethyl sulfoxide
[EMIm][OAc]: 1-ethyl-3-methylimidazolium acetate
EMImS: 1-ethyl-3-methylimidazole-2-thione
EPR: electron paramagnetic resonance
ESI-ToF-MS: electrospray ionization time-of-flight mass spectrometry
h: hour
HMBC: heteronuclear multiple bond correlation
HSQC: heteronuclear single quantum correlation
IL: ionic liquid
min: minute
mol %: mole percent
NaOtBu: sodium *tert*-butoxide
NHC: N-heterocyclic carbene
NIR: near-infrared
nm: nanometer
NMR: nuclear magnetic resonance
Nu: nucleophile
S_8_: sulfur
S_2_N_2_: disulfur dinitride
S_4_N_4_: tetrasulfur tetranitride
(SN)_*x*_: poly(sulfur nitride)
ST: spin trap
UM: ultra marine
UV/vis: ultraviolet–visible
wt %: weight percent
