# Supplementary material for: 1-Ethyl-3-methylimidazolium Acetate as a Reactive Solvent for Elemental Sulfur and Poly(sulfur nitride)
Source: J Phys Chem B. 2024 Jun 1;128(23):5700–12. doi: 10.1021/acs.jpcb.4c01536 (PMC11182232; doi:10.1021/acs.jpcb.4c01536)
Supplement: Supplementary file 1 — jp4c01536_si_001.pdf [file jp4c01536_si_001.pdf]

Supporting Information of

1-Ethyl-3-methylimidazolium acetate as reactive solvent for elemental sulfur and poly(sulfur nitride)

Julian Radicke, Karsten Busse, Vanessa Jerschabek, Haleh Hashemi Haeri, Muhammad Abu Bakar, Dariush Hinderberger, and Jörg Kressler *

Department of Chemistry, Martin Luther University Halle-Wittenberg, von-Danckelmann-Platz 4, D-06120 Halle (Saale), Germany

*Corresponding author

Table of contents

1 NMR spectroscopic study of sulfur and $(SN)_x$ in [EMIm][OAc] 2

1.1 Sample preparation 2

1.2 Integral results of the NMR spectroscopy 3

1.3 NMR spectra of 1-ethyl-3-methylimidazolium acetate [EMIm][OAc] 4

1.4 NMR spectra of sulfur in [EMIm][OAc] with different wt. % 5

1.5 NMR spectra of the EMImS without [EMIm][OAc] 10

1.6 NMR spectra of $(SN)_x$ in [EMIm][OAc] with different wt. % 11

1.7 NMR spectra of the products from the reaction of $(SN)_x$ with [EMIm][OAc].........16

2 UV/VIS spectroscopy 19

3 EPR study of sulfur and $(SN)_x$ in [EMIm][OAc]..22

4 Alternative reaction mechanisms of sulfur with [EMIm][OAc] 23

1 NMR spectroscopic study of sulfur and $(SN)_x$ in [EMIm][OAc]

1.1 Sample preparation

Table S1: Comparison of the weights, weight percentages (wt. %) and molar ratios of sulfur in [EMIm][OAc].

sample [wt. %]$_{theo}$	1.00	2.00	3.00	4.00	5.00*
mass of stock solution [mg]	49.10	81.60	126.40	164.8	-
mass of IL [mg]	157.60	119.30	76.40	32.30	718.9
total mass	206.7	200.9	202.8	197.1	754.4
sample [wt. %]$_{real}$	1.15	1.96	3.01	4.04	4.83
$n(S_8)/n(IL)$ [mol %]	0.70	1.30	2.10	2.80	3.30
$n(S_1)/n(IL)$ [mol %]	6.20	10.60	16.50	22.30	26.20

*stock solution

Table S2: Comparison of the weights, weight percentages (wt. %) and molar ratios of $(SN)_x$ in [EMIm][OAc].

sample [wt. %]$_{theo}$	1.00	1.50	2.00	2.50	3.00[1]
mass of stock solution [mg]	67.70	106.30	143.90	171.70	-
mass of IL [mg]	131.80	100.30	69.20	33.80	1204.30
total mass	199.50	206.60	213.10	205.50	1241.49
sample [wt. %]$_{real}$	1.01	1.54	2.01	2.49	2.99
$n((SN)_x)/n(IL)$ [mol %][1]	0.90	1.50	1.90	2.40	2.90
$n(S_1)/n(IL)$ [mol %]	3.80	5.80	7.70	9.60	11.50

[1]stock solution

[2]we had calculate the $n((SN)_x)$ with the molar mass of S_4N_4 because the repetitive units of $(SN)_x$ were unknown.

1.2 Integral results of the NMR spectroscopy

Table S3: Comparison of the integrals of the ^{1}H NMR spectra of sulfur in IL with different sulfur concentration.

**S in IL**	**intergrals of the protons***							
**[wt. %]**	**H-4/5**	**HS-4/5**	**H-6**	**HS-6**	**H-7**	**HS-7**	**H-8**	**HS-8**
0.00	2.25	0.00	3.21	0.00	2.01	0.00	3.10	0.00
1.15	2.18	0.22	2.90	0.24	2.00	0.20	2.87	0.21
1.96	1.98	0.34	2.67	0.32	1.80	0.25	2.66	0.36
3.01	1.99	0.50	2.44	0.51	1.67	0.39	2.54	0.53
4.04	1.82	0.58	2.31	0.67	1.57	0.51	2.35	0.70
4.83	1.68	0.60	2.16	0.82	1.49	0.57	2.18	0.80

*The manual calibration of the integrals was done by setting the signal of the methyl group of the acetate ion to 3.00 in all spectra.

Table S4: Comparison of the integrals of the ^{1}H NMR spectra of $(SN)_x$ in IL with different $(SN)_x$ concentration.

**$(SN)_x$**	**intergrals of the protons***							
**in IL [wt. %]**	**H-4/5**	**HS-4/5**	**H-6**	**HS-6**	**H-7**	**HS-7**	**H-8**	**HS-8**
0.00	2.25	0.00	3.05	0.00	2.04	0.00	3.02	0.00
1.01	2.11	0.10	2.92	0.16	1.98	0.14	2.88	0.23
1.54	2.06	0.15	2.83	0.21	1.88	0.17	2.76	0.24
2.01	1.99	0.17	2.71	0.23	1.85	0.17	2.74	0.29
2.49	1.95	0.19	2.66	0.30	1.75	0.20	2.63	0.33
2.99	1.85	0.22	2.51	0.32	1.71	0.22	2.54	0.38

*The manual calibration of the integrals was done by setting the signal of the methyl group of the acetate ion to 3.00 in all spectra.

1.3 NMR spectra of 1-ethyl-3-methylimidazolium acetate [EMIm][OAc]

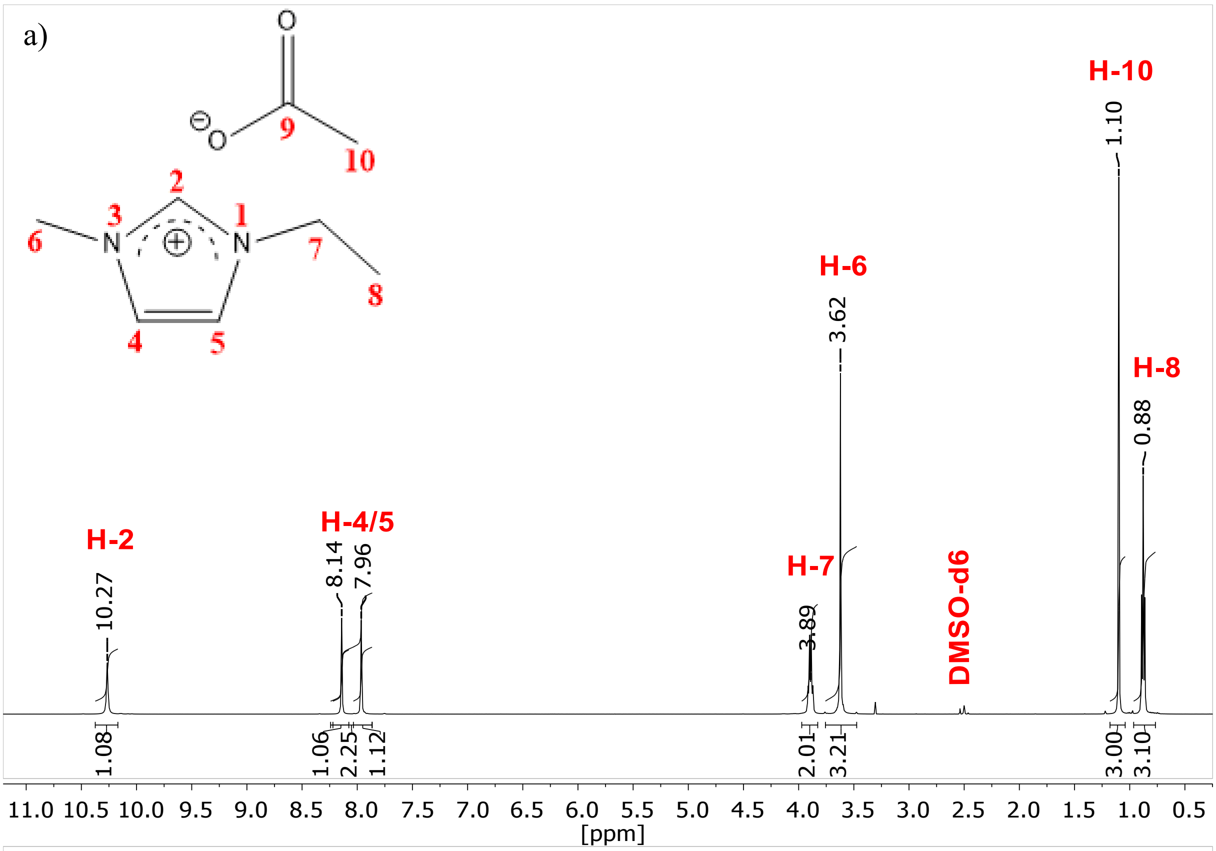

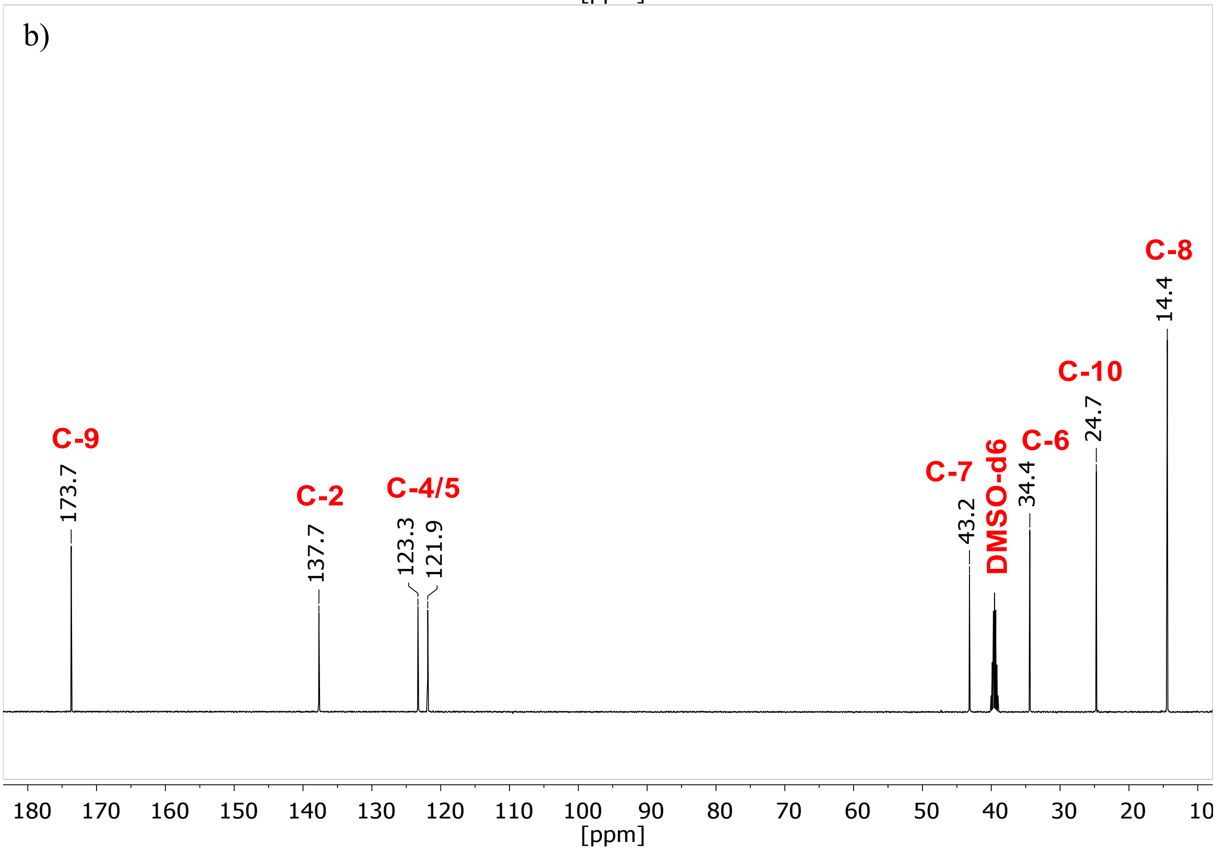

Figure S1: a) The ^{1}H NMR spectrum and b) the ^{13}C NMR spectrum of the IL [EMIm][OAc] in DMSO-d6 at T = 27 °C.

1.4 NMR spectra of sulfur in [EMIm][OAc] with different wt. %

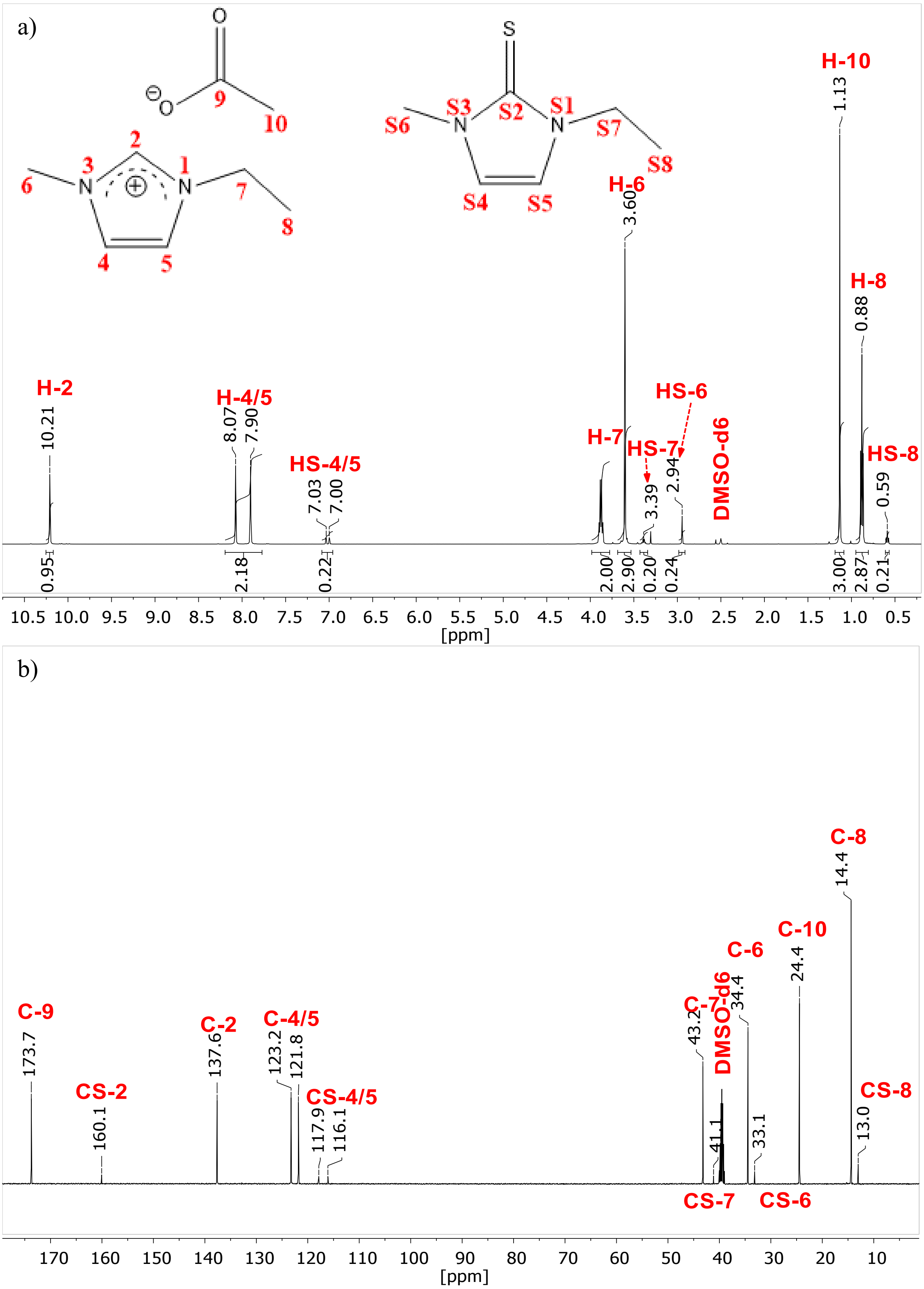

Figure S2: a) The ^{1}H NMR spectrum and b) ^{13}C NMR spectrum of 1.15wt. % sulfur in [EMIm][OAc].

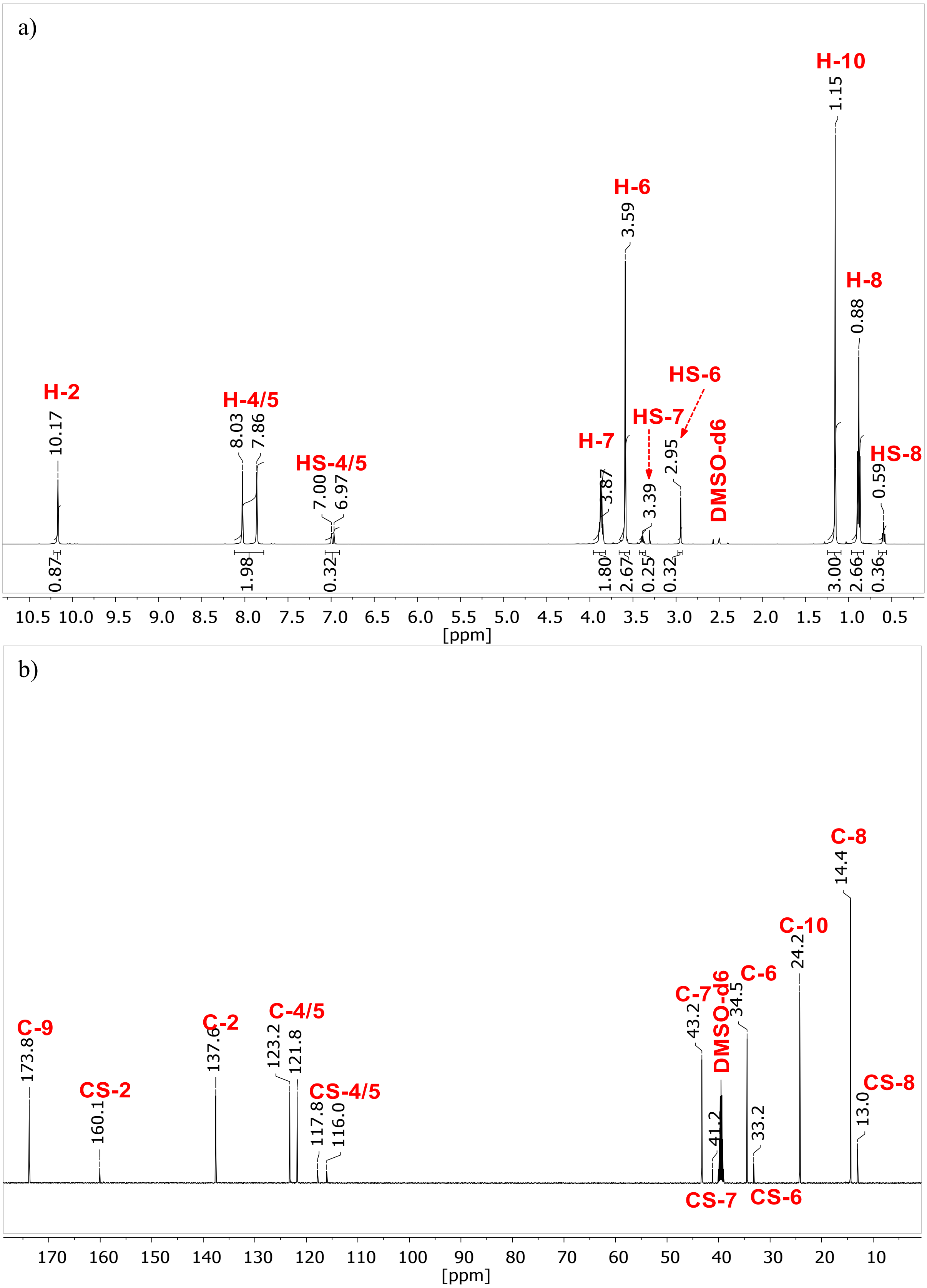

Figure S3: a) The ^{1}H NMR spectrum and b) ^{13}C NMR spectrum of 1.96wt. % sulfur in [EMIm][OAc].

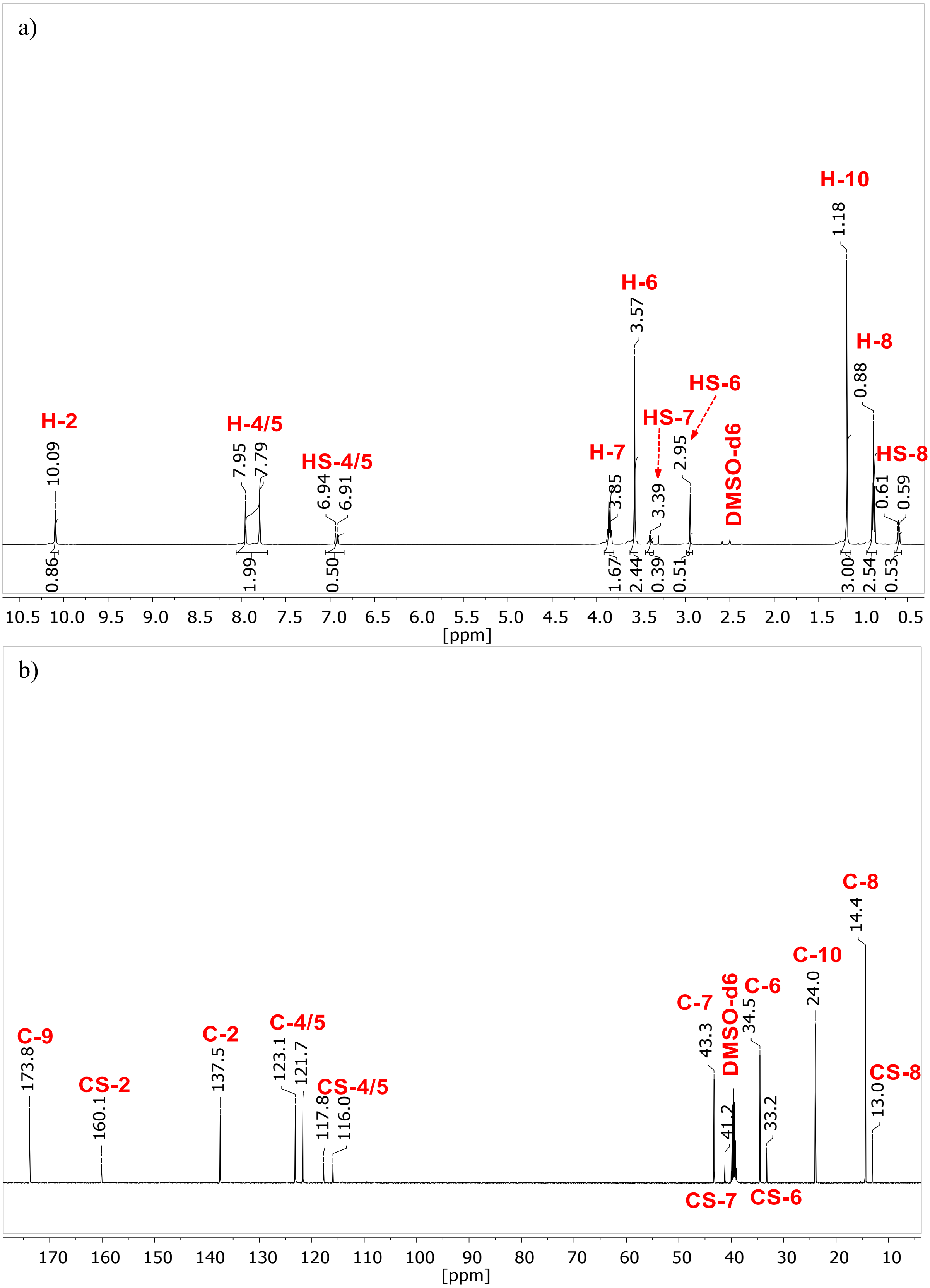

Figure S4: a) The ^{1}H NMR spectrum and b) ^{13}C NMR spectrum of 3.01wt. % sulfur in [EMIm][OAc].

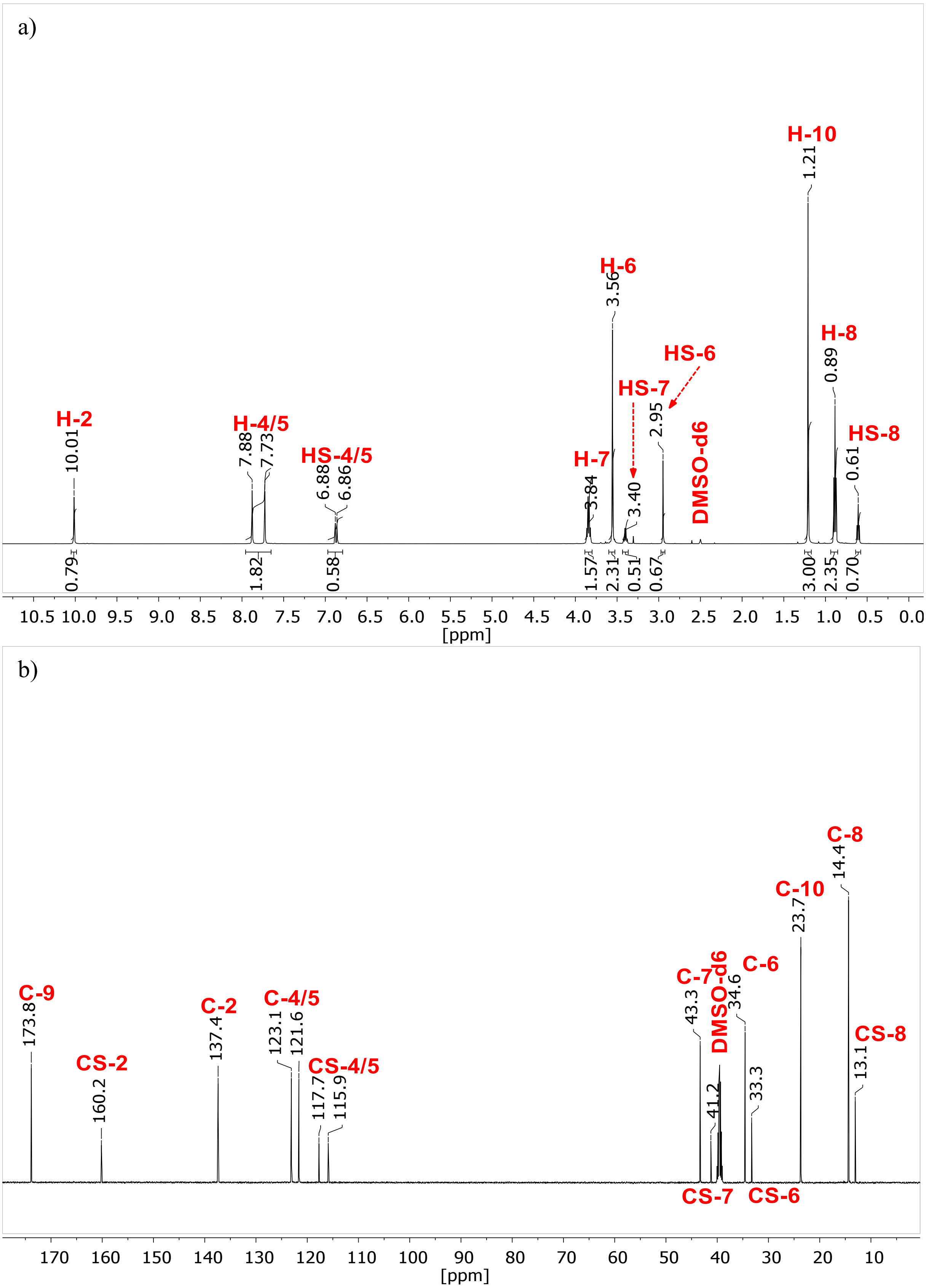

Figure S5: a) The ^{1}H NMR spectrum and b) ^{13}C NMR spectrum of 4.04wt. % sulfur in [EMIm][OAc].

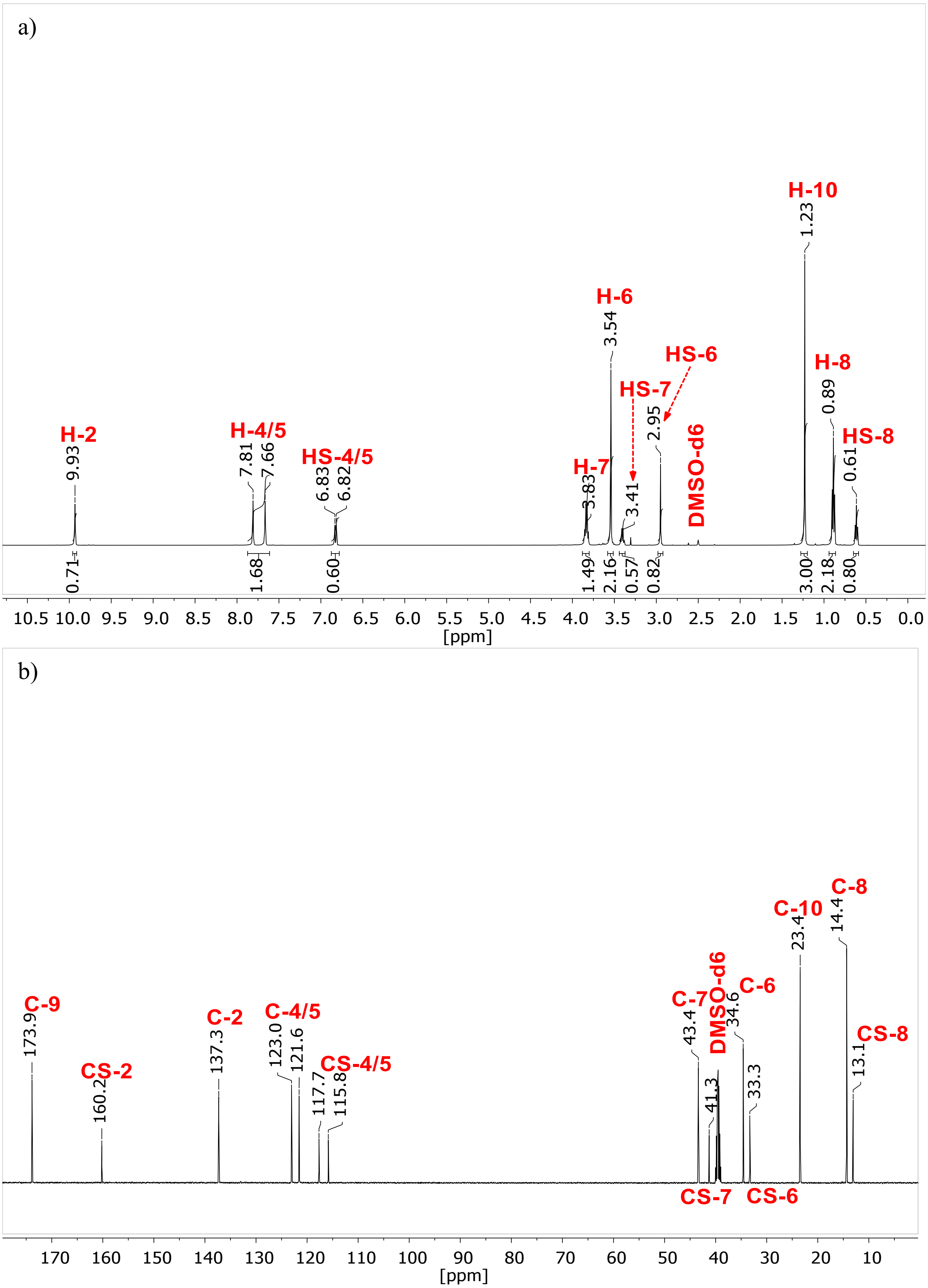

Figure S6: a) The ^{1}H NMR spectrum and b) ^{13}C NMR spectrum of 4.83wt. % sulfur in [EMIm][OAc].

1.5 NMR spectra of the EMImS without [EMIm][OAc]

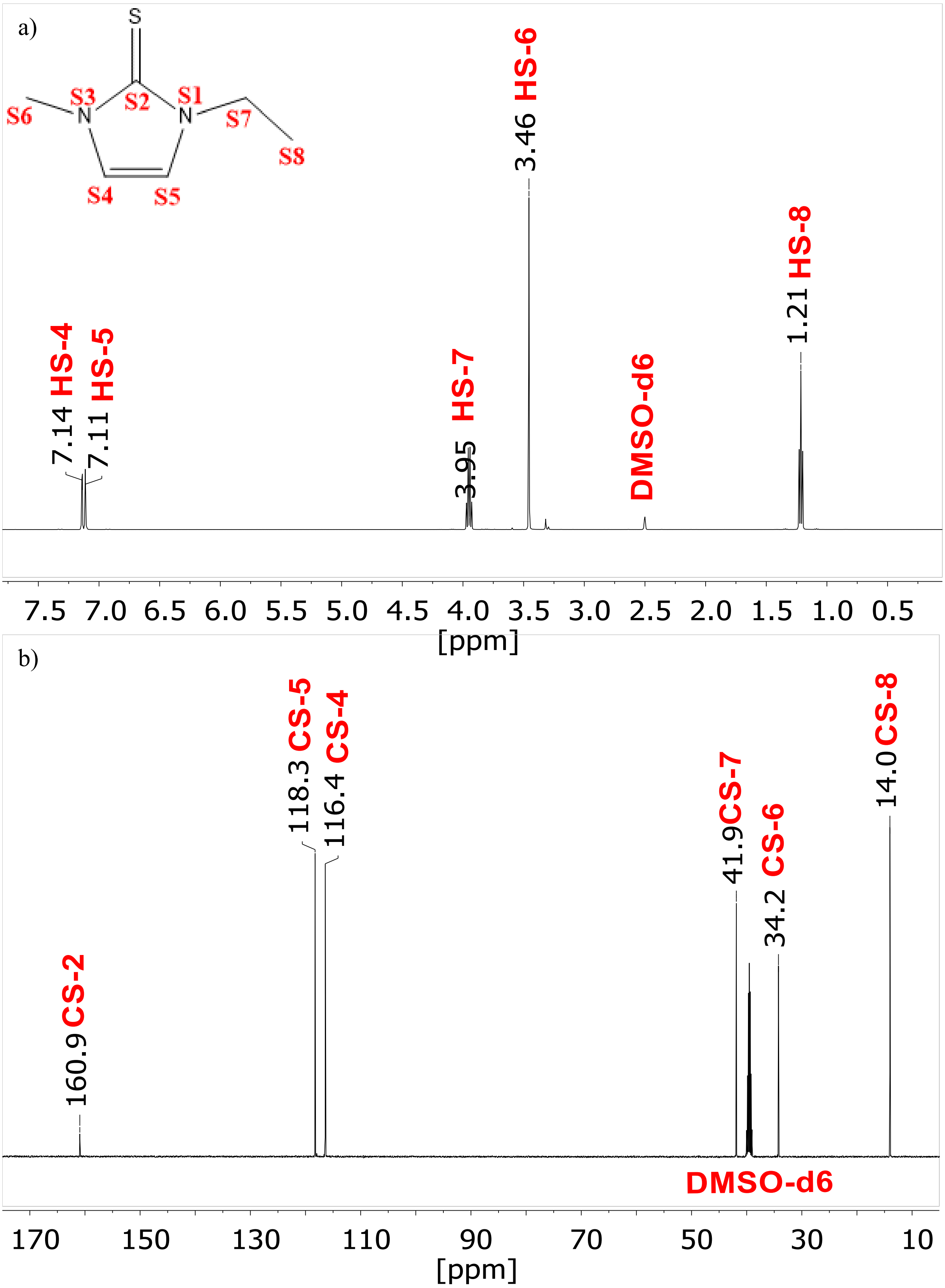

Figure S7: a) The ^{1}H NMR spectrum of the isolated product of sulfur in IL and b) the ^{13}C NMR spectrum of the imidazole thione.

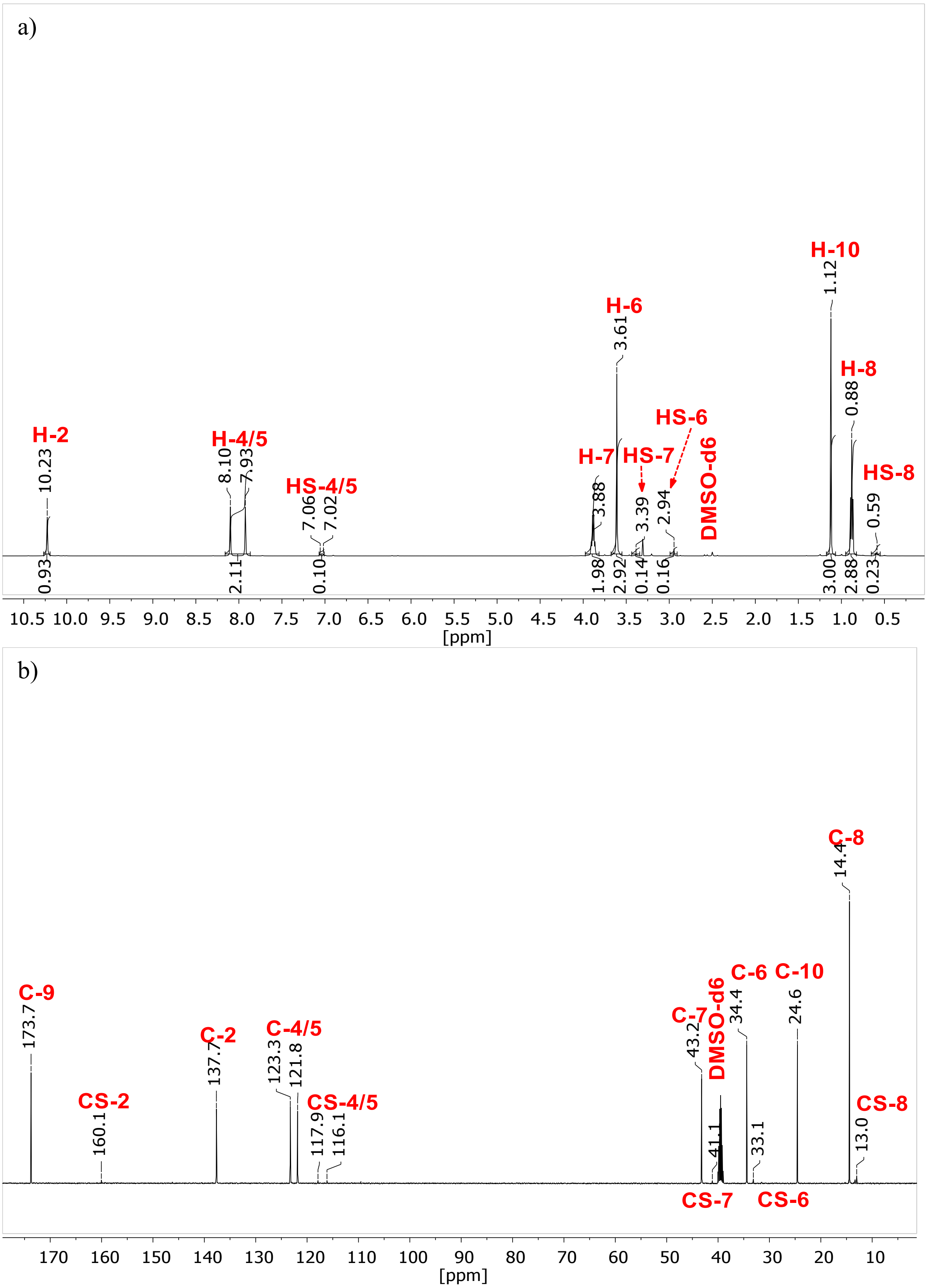

1.6 NMR spectra of $(SN)_x$ in [EMIm][OAc] with different wt. %

Figure S8: a) The ^{1}H NMR spectrum and b) ^{13}C NMR spectrum of 1.01wt. % $(SN)_x$ in [EMIm][OAc].

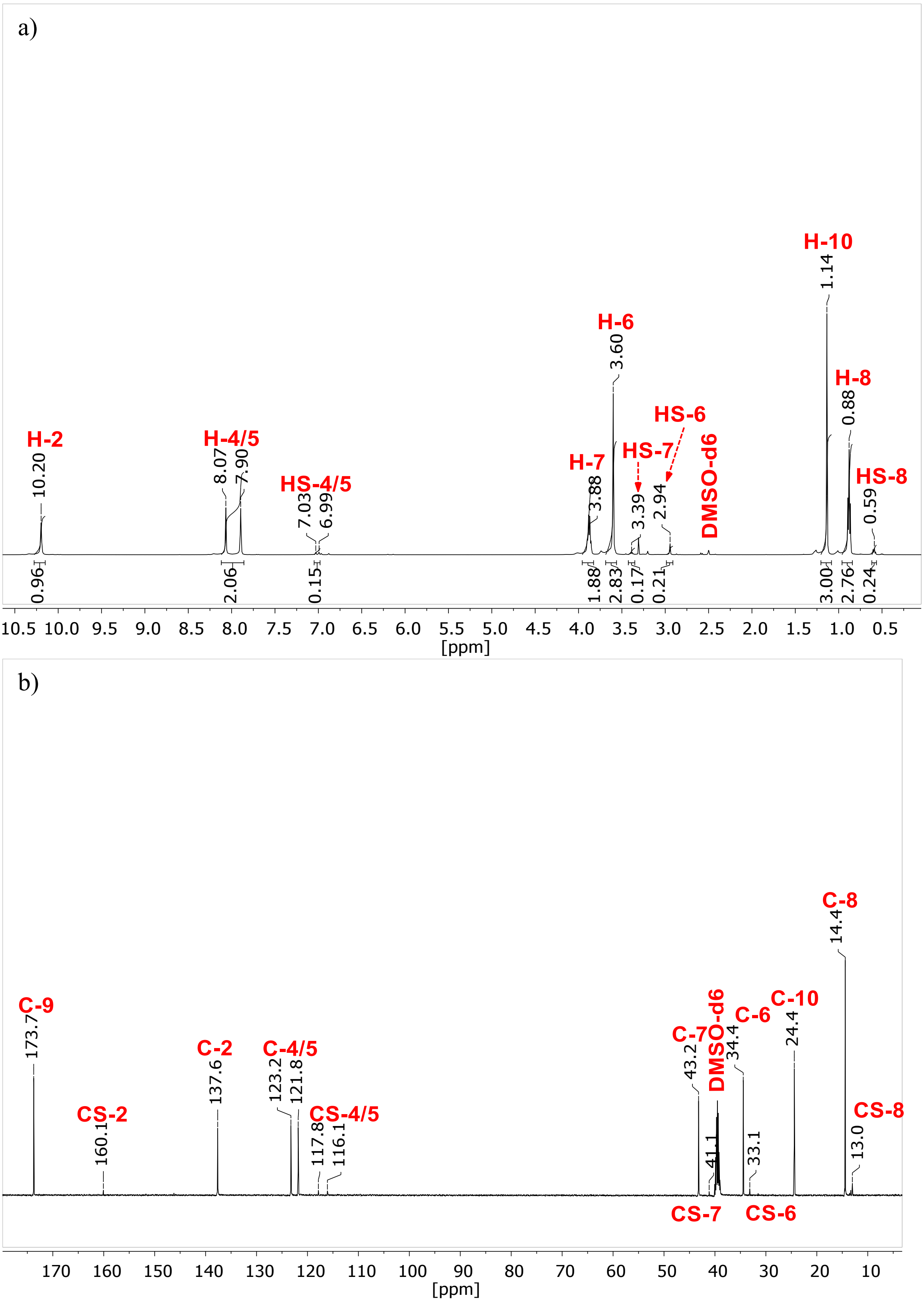

Figure S9: a) The ^{1}H NMR spectrum and b) ^{13}C NMR spectrum of 1.54wt. % $(SN)_x$ in [EMIm][OAc].

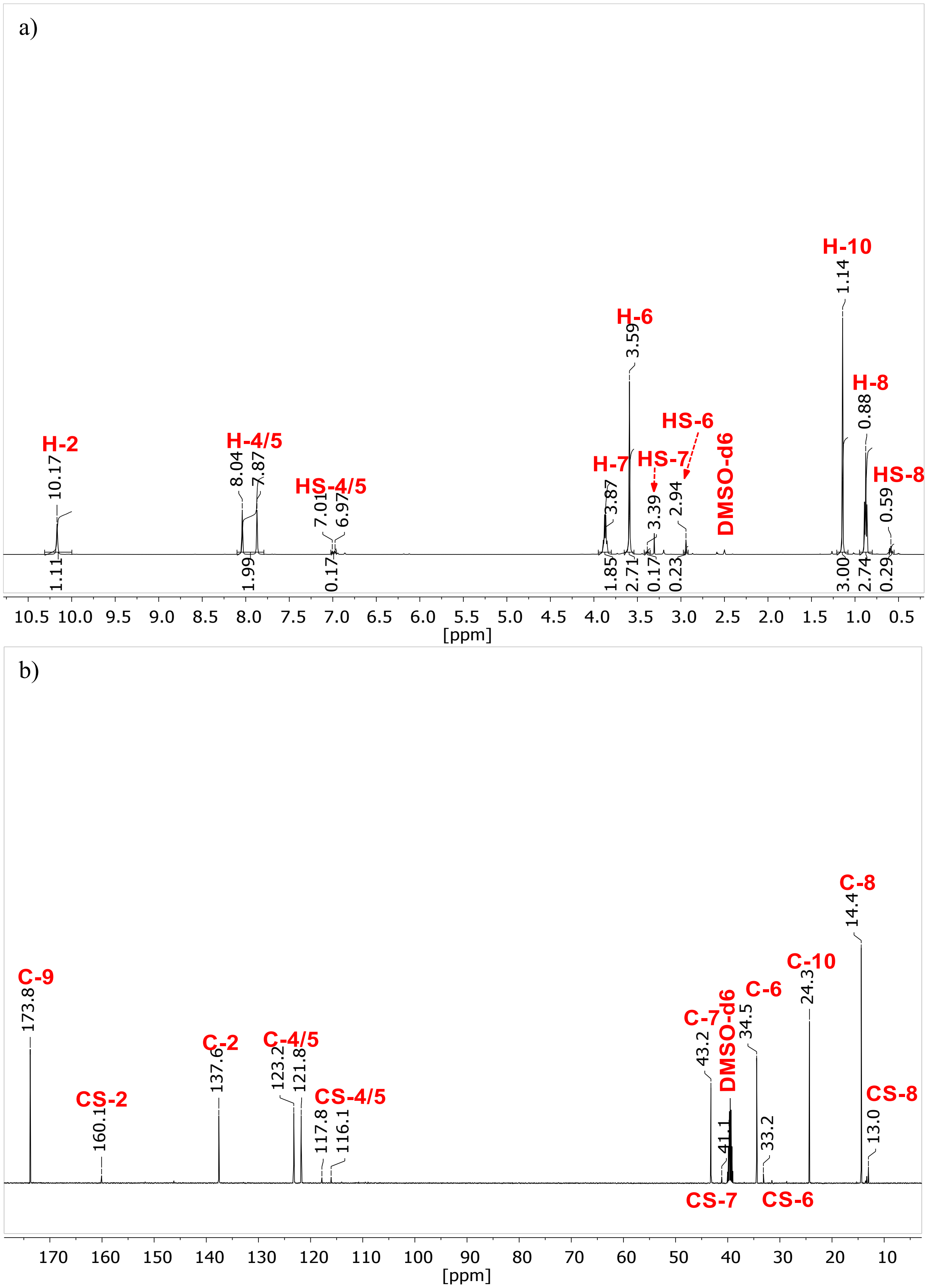

Figure S10: a) The ^{1}H NMR spectrum and b) ^{13}C NMR spectrum of 2.01wt. % $(SN)_x$ in [EMIm][OAc].

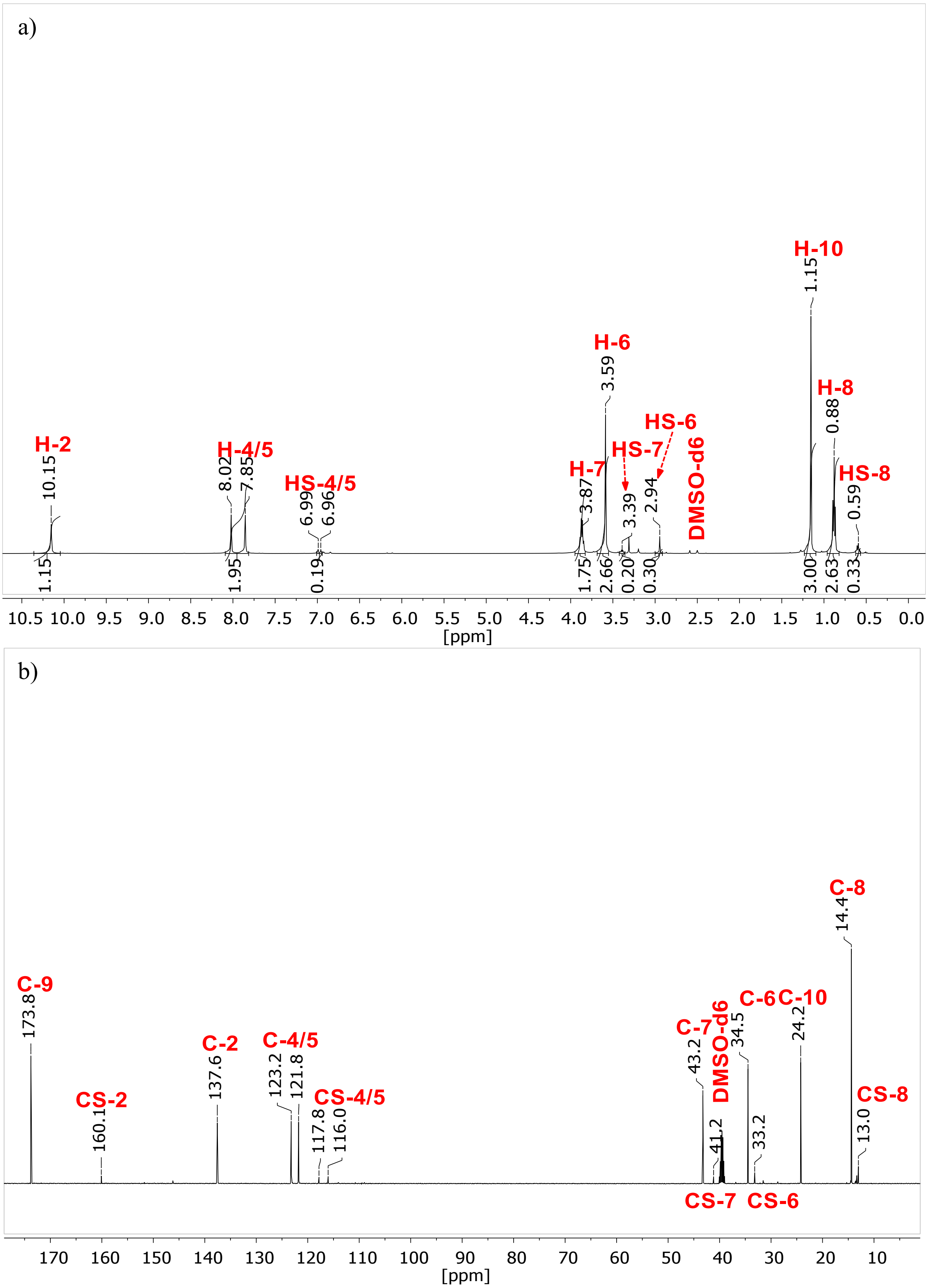

Figure S11: a) The ^{1}H NMR spectrum and b) ^{13}C NMR spectrum of 2.49wt. % $(SN)_x$ in [EMIm][OAc].

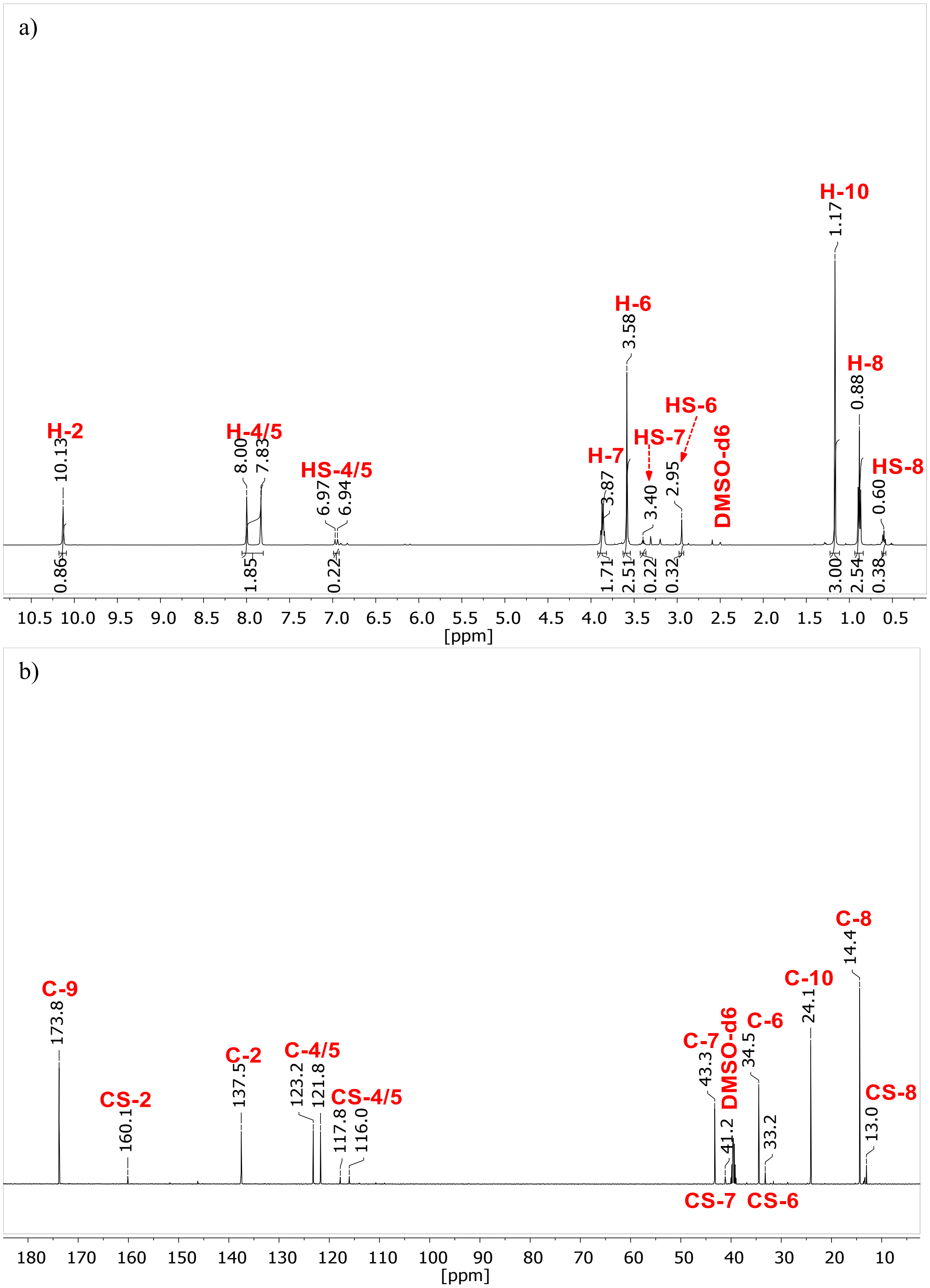

Figure S12: a) The ^{1}H NMR spectrum and b) ^{13}C NMR spectrum of 2.99wt. % $(SN)_x$ in [EMIm][OAc].

1.7 NMR spectra of the products from the reaction of $(SN)_x$ with [EMIm][OAc]

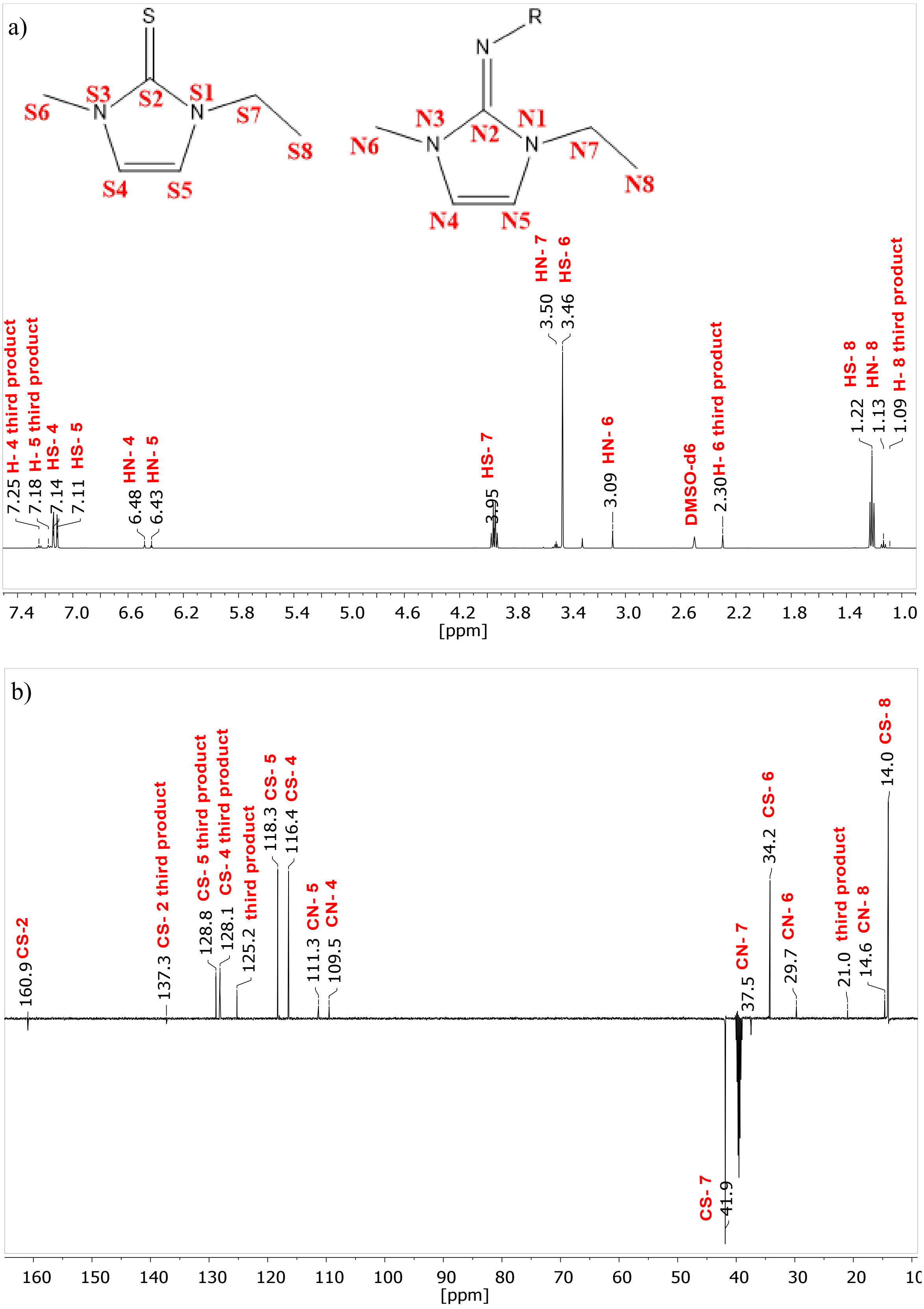

Figure S13: a) The ^{1}H NMR spectrum and b) ^{13}C NMR spectrum of the products of $(SN)_x$ in IL after the cleaning process

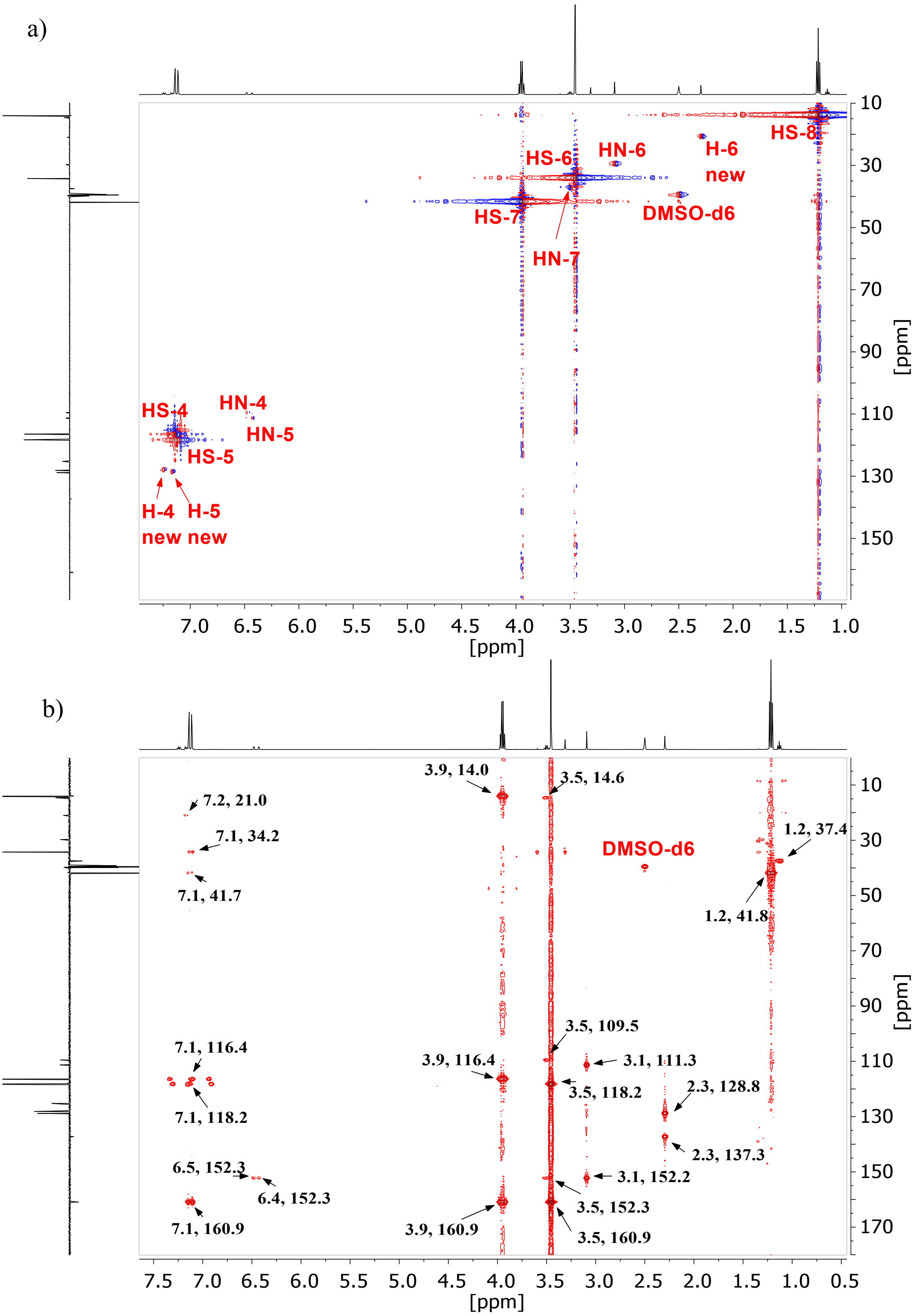

Figure S14: a) The gHSQCAD and b) gHMBCAD NMR spectra of the products of the reaction of $(SN)_x$ in IL after the cleaning process.

Table S5: Comparison of the chemical shifts of sulfur and $(SN)_x$ in IL and of the isolated products.

proton	chemical shift (*multiplet*)* [ppm]				
	[EMIm][OAc]	Sulfur + IL	$(SN)_x$ + IL	Thione (S)	Thione ($(SN)_x$)
H-2	10.27 (*s*)	9.93 (*s*)	10.13 (*s*)	-	-
H-4	8.14 (*s*)	7.81 (*s*)	8.00 (*s*)	-	-
H-5	7.96 (*s*)	7.66 (*s*)	7.83 (*s*)	-	-
HS-4	-	6.83 (*d*)	6.97 (*d*)	7.14 (*d*)	7.14 (*d*)
HS-5	-	6.82 (*d*)	6.94 (*d*)	7.11 (*d*)	7.11 (*d*)
HN-4	-	-	6.16 (*d*)	-	6.48 (*d*)
HN-5	-	-	6.10 (*d*)	-	6.43 (*d*)
H-6	3.62 (*s*)	3.54 (*s*)	3.58 (*s*)	-	-
HS-6	-	2.95 (*s*)	2.95 (*s*)	3.46 (*s*)	3.46 (*s*)
HN-6	-	-	-	-	3.09 (*s*)
H-7	3.89 (*q*)	3.83 (*q*)	3.87 (*q*)	-	-
HS-7	-	3.41 (*q*)	3.40 (*q*)	3.95 (*q*)	3.95 (*q*)
HN-7	-	-	-	-	3.50 (*q*)
H-8	0.88 (*t*)	0.89 (*t*)	0.88 (*t*)	-	-
HS-8	-	0.61 (*t*)	0.60 (*t*)	1.21 (*t*)	1.22 (*t*)
HN-8	-	-	0.51 (*t*)	-	1.13 (*t*)

**s* = singlet; *d* = doublet; *q* = quartet; *t* = triplet

Table S6: Comparison of the molar ratio of sulfur to IL and the sum of the integral of thione to IL in different weight percent [wt. %].

Sulfur in IL [wt. %]	Molar ratio of sulfur/IL and NMR integrals ratio of HS/IL	
	$n(S_1)/n(IL)$ [mol %]	I(HS)/I(IL) [mol %]
1.15	0.062	0.063
1.96	0.106	0.106
3.01	0.165	0.165
4.04	0.223	0.223
4.83	0.262	0.262

Table S7: Comparison of the ratios of $(SN)_x$ to IL and the sum of the integrals of HS to IL in different weigth percent [wt. %].

$(SN)_x$ in IL [wt. %]	Molar ratio of $(SN)_x$/IL and NMR integrals ratio of HS/IL	
	$n(S_1)/n(IL)$ [mol %]	I(HS)/I(IL) [mol %]
1.01	0.038	0.032
1.54	0.058	0.054
2.01	0.077	0.073
2.49	0.096	0.088
2.99	0.115	0.107

2 UV/VIS spectroscopy

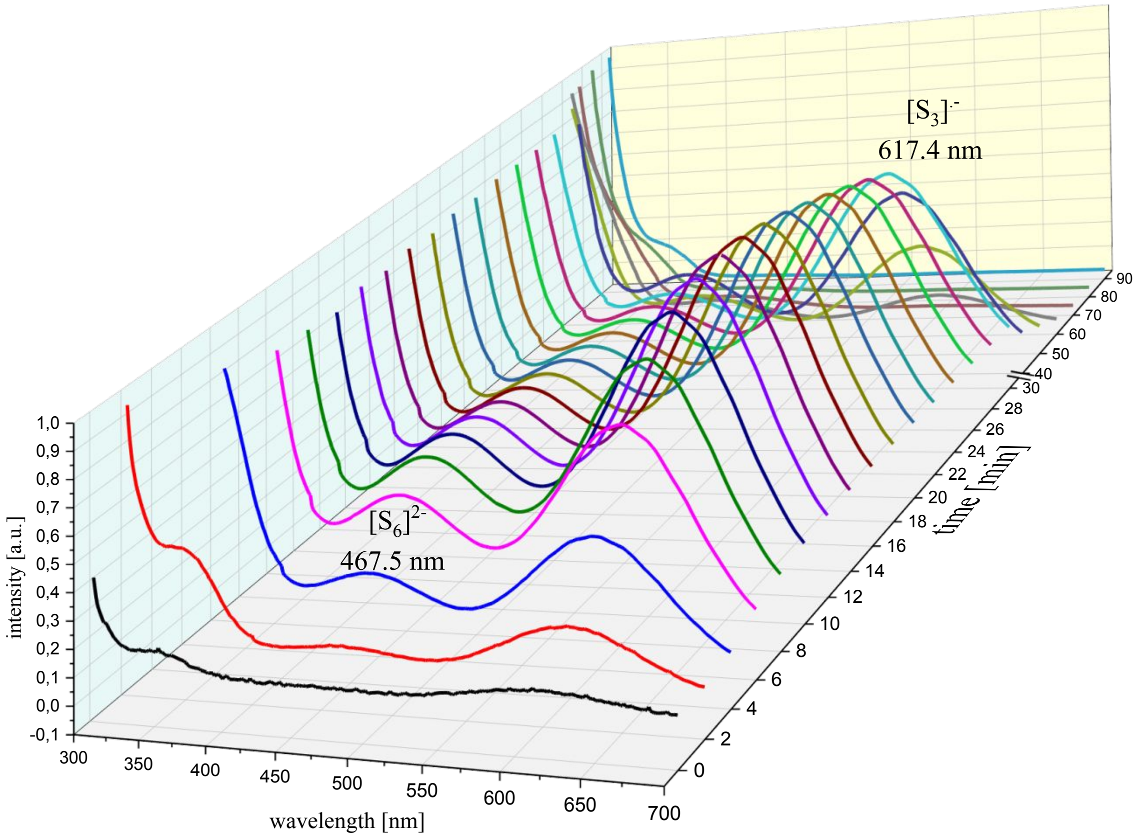

Figure S15: *UV/VIS spectra of sulfur (1.0 mg) in IL (100 µL) and DMSO (2 mL) over the time.*

Kinetic investigation of $[S_8]$ decay:

We assume to have the reaction scheme in high excess of IL:

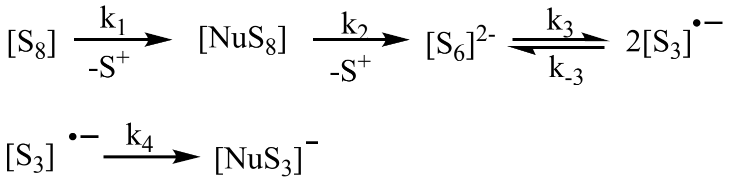

The abbreviation $[NuS_8]$ describes the intermediate between $[S_8]$ and $[S_6]^{2-}$, for which the molecular structure cannot be exactly identified. Also $[NuS_3]^{-}$ describes the degradation product of $[S_3]^{\cdot-}$, which was also not identified in detail.

$$\frac{d}{dt}[S_8] = -k\,[IL][S_8] = -\,k_1[S_8] \quad \text{(S1)}$$

$$\frac{d}{dt}[NuS_8] = k\,[IL][S_8] - k\,[IL][NuS_8] = k_1[S_8] - k_2[NuS_8] \quad \text{(S2)}$$

$$\frac{d}{dt}[S_6]^{2-} = k\,[IL][NuS_8] - k_3[S_6]^{2-} + k_{-3}([S_3]^{\cdot-})^2 = k_2[NuS_8] - k_3[S_6]^{2-} + k_{-3}([S_3]^{\cdot-})^2 \quad \text{(S3)}$$

$$\frac{d}{dt}[S_3]^{\cdot-} = 2k_3[S_6]^{2-} - 2k_{-3}([S_3]^{\cdot-})^2 - k_4[S_3]^{\cdot-} \quad \text{(S4)}$$

$$\frac{d}{dt}\left([S_6]^{2-} + [S_3]^{\cdot-}/2\right) = k_2[NuS_8] + \frac{k_4}{2}[S_3]^{\cdot-} \approx k_2[NuS_8] + k_5\left([S_6]^{2-} + [S_3]^{\cdot-}/2\right) \quad \text{(S5)}$$

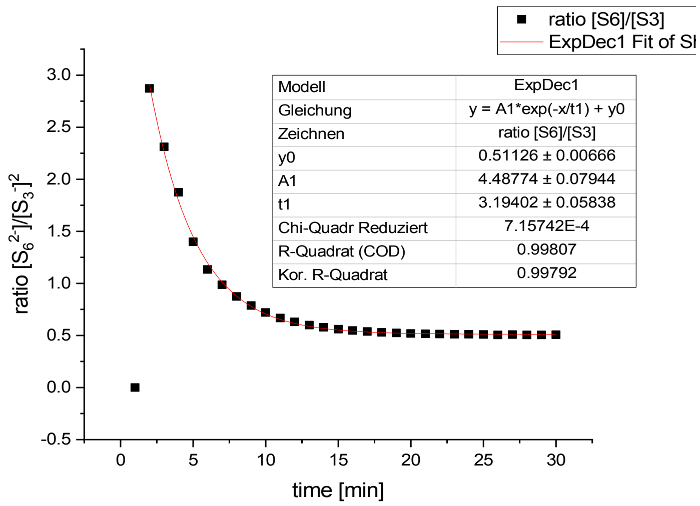

Figure S16: *Plot of the ratio of $[S_6]^{2-}$ to $[S_3]^{\cdot-}$ over the time. The formation of $[S_6]^{2-}$ starts first and the equilibrium between both components is reached after ~10 min.*

The solutions of the kinetic equations are:

$$[S_8] = [S_8]_0 e^{-k_1 t} \tag{S6}$$

$$[NuS_8] = k_1[S_8]_0\left(\frac{(e^{-k_1 t} - e^{-k_2 t})}{(k_2 - k_1)}\right) \qquad (k_2 \neq k_1) \tag{S7}$$

$$[NuS_8] = k_1[S_8]_0 t e^{-k_1 t} \qquad (k_2 = k_1) \tag{S8}$$

$$\left([S_6]^{2-} + [S_3]^{\cdot -}/2\right) = \frac{k_1 k_2 [S_8]_0}{(k_2 - k_1)}\left(\frac{e^{-k_1 t}}{(k_5 - k_1)} - \frac{e^{-k_2 t}}{(k_5 - k_2)} - \left(\frac{1}{(k_5 - k_1)} - \frac{1}{(k_5 - k_2)}\right)e^{-k_5 t}\right) \tag{S9}$$

$$\left([S_6]^{2-} + [S_3]^{\cdot -}/2\right) = \frac{{k_1}^2 [S_8]_0}{(k_5 - k_1)^2}\left(e^{-k_5 t} + e^{-k_1 t}(t(k_5 - k_1) - 1)\right) \qquad (k_2 = k_1) \tag{S10}$$

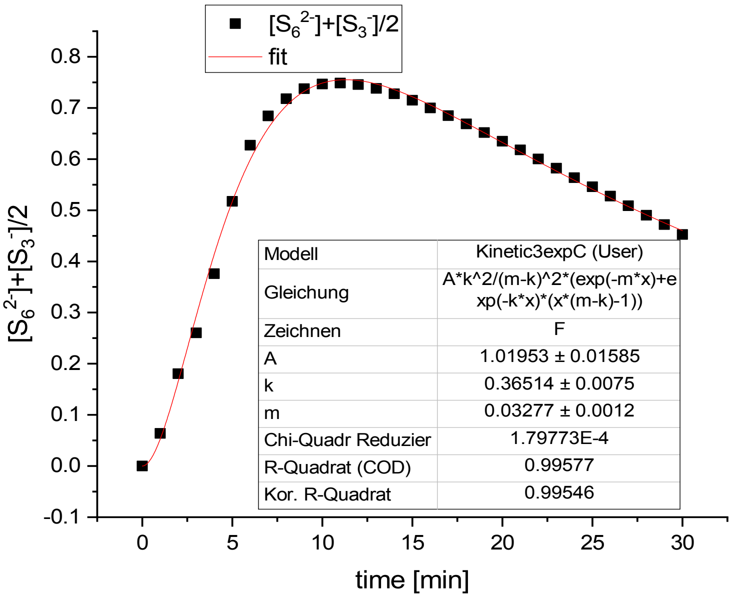

Figure S17: Plot of $[S_6]^{2-}$ + $[S_3]^{\cdot -}/2$ during time with fit according to eq. S10 showing the degradation of $[S_3]^{\cdot -}$.

3 EPR study of sulfur and $(SN)_x$ in [EMIm][OAc]

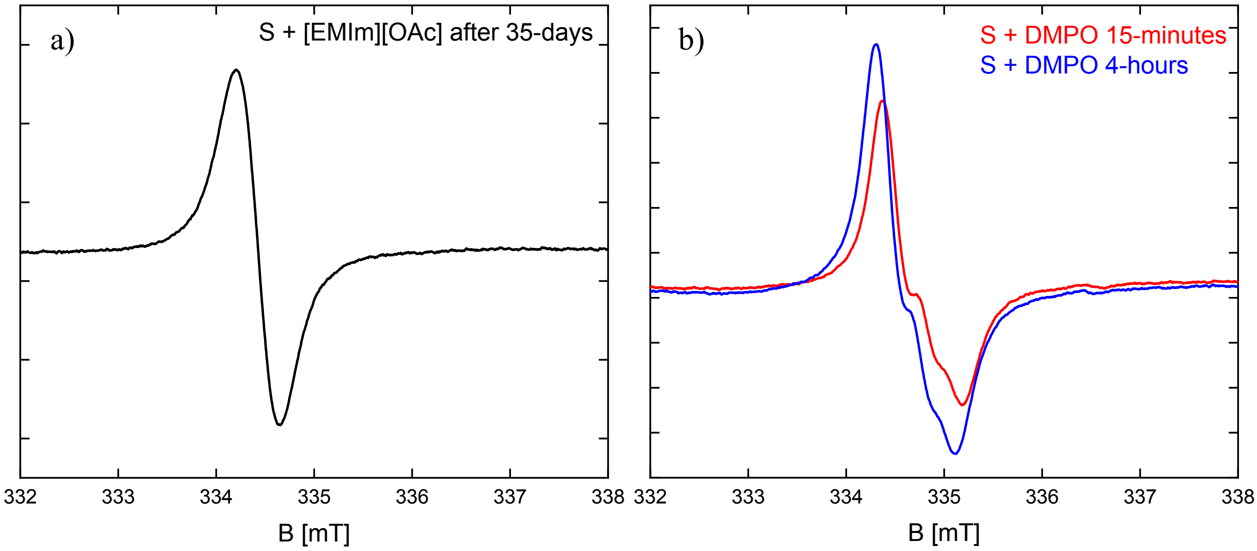

Figure S18: a) EPR spectrum of sulfur in IL after 35 days and b) sulfur with DMPO in IL.

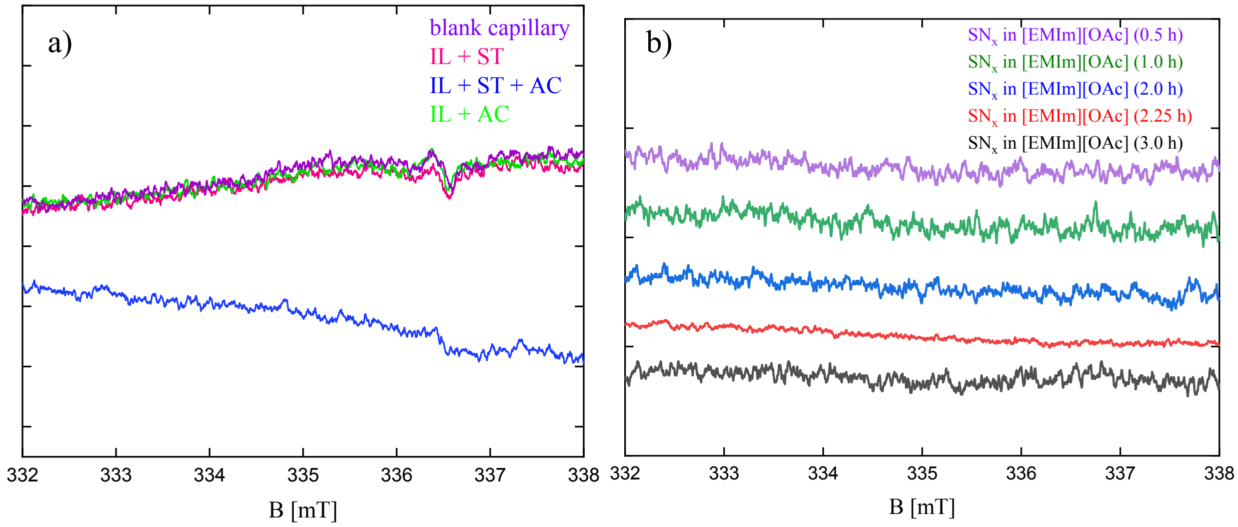

Figure S19: a) EPR reference spectra and b) EPR spectra of $(SN)_x$ in [EMIm][OAc] without spin trap.

Table S8: EPR measurements values of sulfur in IL over the time.

Time [h]	B [mT]	ν [GHz]	g-value	B_{max} [mT]	B_{min} [mT]	dB [mT]
0.75	334.74	9.42810	2.01235	334.493	334.996	0.503
1.50	334.73	9.42807	2.01243	334.496	334.957	0.461
2.00	334.71	9.42782	2.01247	334.462	334.969	0.507
2.50	334.69	9.42767	2.01255	334.458	334.929	0.471
3.00	334.68	9.42756	2.01263	334.436	334.913	0.477
3.50	334.67	9.42748	2.01265	334.419	334.901	0.482
4.00	334.66	9.42746	2.01272	334.427	334.888	0.461
4.50	334.65	9.42743	2.01275	334.418	334.891	0.473
5.00	335.01	9.42738	2.01059	334.408	334.876	0.468
5.50	334.64	9.42738	2.01282	334.406	334.868	0.462
6.00	334.63	9.42734	2.01284	334.394	334.867	0.473
6.50	334.63	9.42734	2.01286	334.400	334.865	0.465
28.00[1]	334.68	9.42866	2.01285	334.476	334.878	0.402
240[2]	334.75	9.42781	2.01224	334.516	334.982	0.466

[1]*sample heated at T = 60 °C*

[2]*measuremend was determined after 10 days*

4 Alternative reaction mechanisms of sulfur with [EMIm][OAc]

Reaction step II':

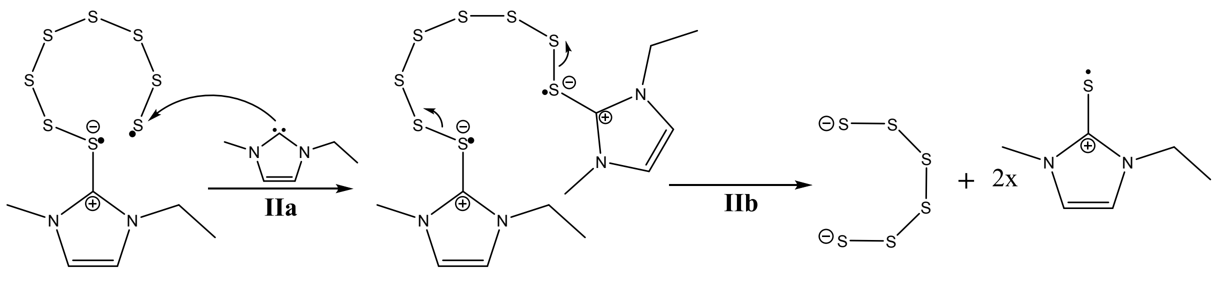

The last sulfur is attacked by the next carbene (**IIa**) (Schmidt pathway)[1], produce an intermediate. In step **IIb** sulfur atoms transmitted the negative charges on the neighbor sulfur atoms and generate a $[S_6]^{2-}$.

Reaction step II'':

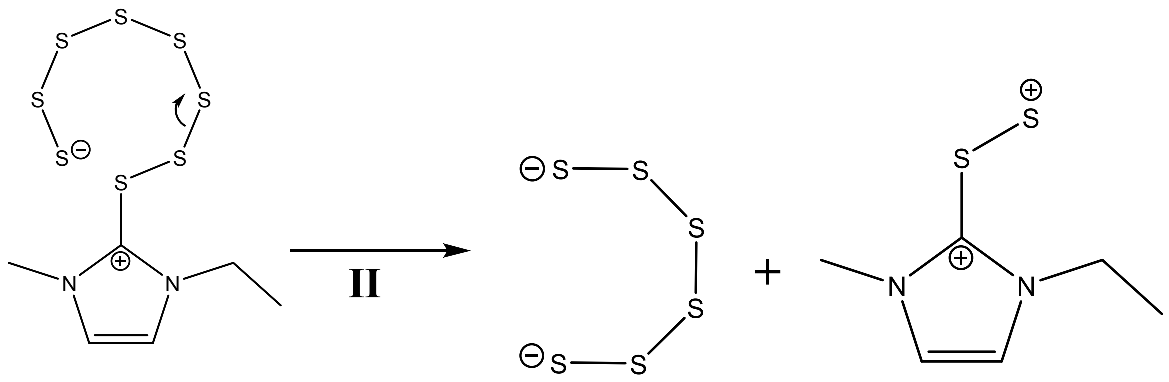

This intermediate generates a positive charge on the second sulfur and dissociates to $[S_6]^{2-}$ and a disulfide-imidazole compound. The next carbene attack the disulfide-imidazole product, generate a disulfide-diimidazole intermediate which generate two sulfur-imidazole radical cations. This reaction scheme would also agree with the observed kinetics.

Reaction step VII':

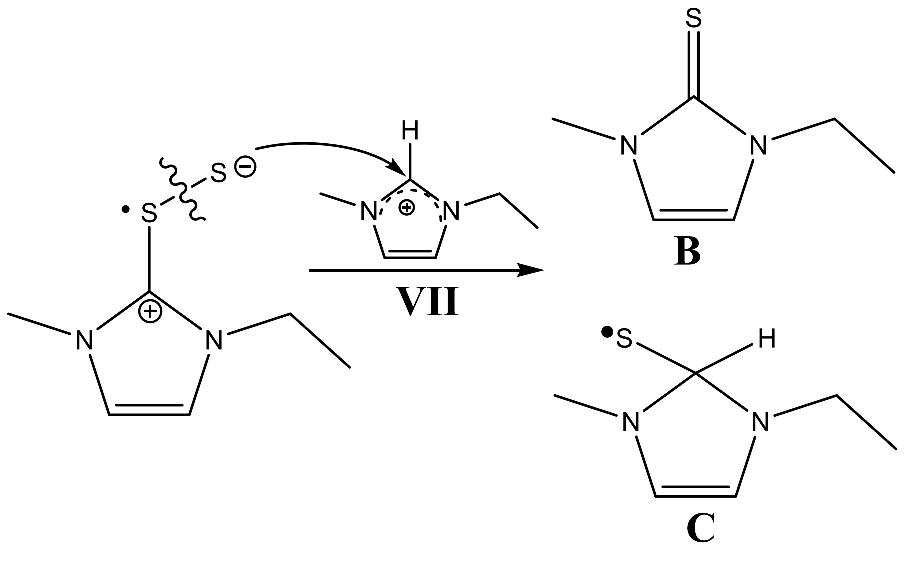

The disulfide-imidazole intermediate from step 6 is attacked by a positive charged imidazole and another radical species **C** is formed. Although the attack by a cation might be favorable, species C was not detected in NMR studies.

References

[1] Sharma, J.; Champagne, P. A. *Mechanisms of the Reaction of Elemental Sulfur and Polysulfides with Cyanide and Phosphines*, *Chemistry* **2023**, *29*, e202203906.
